# Immunogenicity and safety of Institut Pasteur du Cambodge (IPC) and updated Thai Red Cross (UTRC) intradermal rabies vaccine regimens among healthy adults in India: A phase 4, open label, equivalence randomised controlled trial

**DOI:** 10.1371/journal.pmed.1005079

**Published:** 2026-10-29

**Authors:** Jeromie Wesley Vivian Thangaraj, Padinjaremattathil Thankappan Ullas, Balaji Dagadu More, Lokesh Shanmugam, Saravanakumar Velusamy, Dinesh Gopal, Subashri Ramamoorthy, Kanagasabai Kaliaperumal, Mugunthan Narayanan Muthulakshmi, Sandhya Arunachalam, Ramaprabha Ramachandran, Gopinath Ramakrishnan, Suresh Arunachalam, Vijay Vidhyakar Jothi Mani, Ganeshnee Anthuvan Valli, Siddharth Pugalendhi, Sabarinathan Ramasamy, Karunakaran Thiyagarajan, Mohamed Hanifah, Sam Joy, Anoop Velayudhan, Suganya Egambaram, Ala Saritha, Manju Rahi, Simmi Tiwari, Vijay Parashramji Bondre, Manoj V. Murhekar, the Rabies Vaccine Immunogenicity Trial (RVIT) study group

**Affiliations:** 1 Department of Infectious Disease Epidemiology, ICMR National Institute of Epidemiology, Chennai, India; 2 Rabies Laboratory, Encephalitis Group, ICMR National Institute of Virology, Pune, India; 3 Clinical Trial and Training Centre, Mahatma Gandhi Medical College and Research Institute (MGMCRI), Pondicherry, India; 4 Communicable Diseases Division, Indian Council of Medical Research, New Delhi, India; 5 Department of Epidemiology, ICMR Vector Control Research Centre, Pondicherry, India; 6 Division of Zoonotic Diseases Programme, National Centre for Disease Control, Directorate General of Health Services, Ministry of Health and Family Welfare, New Delhi, India; University of Bern: Universitat Bern, SWITZERLAND

## Abstract

**Background:**

Shorter rabies post-exposure prophylaxis (PEP) regimens could improve compliance and access by reducing the number of clinic visits and vaccine requirement per patient. We compared the immunogenicity and safety of a 1-week intradermal Institut Pasteur du Cambodge (IPC) regimen with a 1-month intradermal Updated Thai Red Cross regimen (UTRC) using two rabies vaccines widely used in India: Purified Chick Embryo Cell vaccine (PCECV) and the Purified Vero Cell Rabies vaccine (PVRV).

**Methods and findings:**

We conducted a phase 4, single-centre, open-label randomised controlled equivalence trial among adult healthy volunteers. Participants were block randomised (1:1:1:1) to receive either the IPC (Days 0, 3, 7) or UTRC (Days 0, 3, 7, 28) intradermal regimen with either PCECV or PVRV. Rabies virus neutralising antibody (RVNA) titres were measured up to 1 year. The primary objective of the trial was to determine the immunogenicity of the Intradermal abridged Cambodia regimen with updated intradermal Thai red cross regimen using two different vaccine types among healthy volunteers in India. The primary outcome was geometric mean concentration ratios (GMR) of RVNA on day 42, assessed separately for each vaccine type. Equivalence for each vaccine was predefined as the entire 95% confidence interval for GMR lying between 0.72 and 1.04. The secondary outcomes included proportion of participants with RVNA titres >= 0.5 IU/mL on Days 14, 28, 42, 90, 180, and 365 and vaccine safety. Between October 10, 2023, and December 23, 2023, 400 participants were randomly assigned (100 per group) to PCECV-IPC, PCECV-UTRC, PVRV-IPC, and PVRV-UTRC. 362 (90.5%) completed PEP regimen and blood draw on day 42. In the prespecified modified intention-to-treat analysis, the Day 42 GMR (IPC vs UTRC) was 0.59 (95% CI: 0.47–0.75) for PCECV and 0.76 (95% CI: 0.61–0.95) for PVRV, which did not meet the prespecified equivalence criterion. All participants, irrespective of baseline serostatus, in four groups had RVNA titres ≥0.5 IU/mL at every assessment from day 14–1 year. Adverse events were similar between regimens and mostly mild. There are important limitations in this study. Nearly one-quarter of participants had baseline RVNA titres ≥0.5 IU/mL despite exclusion of participants with a reported history of rabies vaccination or dog bite, suggesting unrecognised prior immunological priming that may have influenced GMC estimates. As the trial was conducted among healthy volunteers rather than people receiving PEP after animal bites, the findings may not fully reflect vaccine performance in routine post-exposure settings.

**Conclusion:**

The IPC regimen did not meet the prespecified equivalence criterion compared to UTRC. All participants achieved RVNA titres ≥0.5 IU/mL indicative of seroprotection from day 14 till 1 year. Policy decisions regarding replacement of UTRC should consider these immunogenicity findings together with compliance, cost, and vaccine availability.

**Trial Registration:**

The trial was registered with the Clinical Trial Registry – India (CTRI/2023/10/058418). URL: https://ctri.nic.in/Clinicaltrials/pmaindet2.php?EncHid=OTAxMDA=&Enc=&userName=).

## Author summary

**Why was this study done?:**

- World Health Organisation recommends the 1-week (three-session) intradermal Institut Pasteur du Cambodge (IPC) regimen for post-exposure prophylaxis (PEP) following category II and III animal bites based on observational studies from Cambodia.
- India and several countries continue to use the updated Thai Red Cross (UTRC) intradermal regimen, which consists of four sessions administered over 1 month.
- Evidence from randomised controlled studies comparing these regimens using Purified Chick Embryo Cell Vaccine (PCECV) and Purified Vero Cell Rabies Vaccine (PVRV), both widely used in India, was needed to inform decisions about adopting shorter regimen.

**What did the researchers do and find?:**

- We randomised 400 healthy adult volunteers to receive either the 1-week IPC or 1-month UTRC regimen, using either PCECV or PVRV.
- IPC regimen produced lower antibody levels than the UTRC regimen, and did not meet the predefined criterion for equivalence.
- Nevertheless, all participants receiving the IPC regimen achieved rabies virus neutralising antibody (RVNA) titres of *≥*0.5 IU/mL by day 14, and maintained throughout 1 year.
- The side effects were mild and similar between the regimens.

**What do these findings mean?:**

- The 1-week IPC regimen did not meet the prespecified equivalence criterion with the 1-month UTRC regimen for antibody concentrations at Day 42, with lower antibody levels observed with the IPC regimen.
- All IPC recipients with evaluable samples had RVNA concentrations of ≥0.5 IU/mL from Day 14–1 year of follow-up. These findings may inform policy decisions about adopting IPC regimen, alongside its potential to reduce cost and improve completion of PEP.
- Because the study was conducted in healthy adult volunteers rather than dog-bite patients, results may not fully reflect real-world vaccine performance in routine post-exposure care.

## Abstract 3

In a phase 4 equivalence trial, Jeromie Thangaraj and colleagues compare the immunogenicity and safety of a one-week versus a one-month rabies vaccine regimen.

## Introduction

Rabies is a fatal, viral zoonotic disease causing an estimated 59,000 annual human rabies deaths globally. India accounts for about one third of the global burden of human rabies deaths [1]. In India, around 9.1 million animal bites and 5726 human deaths due to dog-mediated rabies are estimated to occur every year [2]. Effective post-exposure prophylaxis (PEP), including wound care, timely and complete anti-rabies vaccination, and passive antibody administration for category 3 wounds, can prevent the onset of symptoms and death. However, in India, only 40% of patients with dog bites complete the prescribed vaccination schedule, despite 80% receiving at least one dose of the vaccine [2]. Studies have shown poor compliance with longer regimens and highlighted the need for shorter anti-rabies vaccination regimens to improve compliance [3–5].

PEP against rabies is safe and effective and has been used by millions worldwide post the development of modern cell-culture derived rabies vaccine and embryonated egg-based vaccines (CCEEVs) [6,7]. For decades, the traditional intramuscular regimen consisting of five doses extending over a 1-month period was the standard approach with CCEEVs. However, high costs and inadequate supply were major limiting factors in making it available at all health facilities, especially in Asia and Africa [8].

Studies have demonstrated that intradermal administration of CCEEVs is safe, equally immunogenic, and cost-effective [9–11]. Considering the advantages of intradermal vaccination, the WHO recommended the use of intradermal rabies vaccination (IDRV) in 1992 [12]. The Oxford regimen introduced in 1984 consisted of giving 8 intradermal doses (each dose 0.1 ml) on Day 0, 4 doses on Day 7, and one dose each on Days 28 and 90 (8-0-4-0-1-1). This regimen was later replaced with the Thai Red Cross (TRC) regimen which consists of two intradermal doses on Days 0, 3, and 7 and one dose each on Days 28 and 90 (2-2-2-0-1-1) [13]. The TRC regimen exhibited low compliance at the Day 90 visit, and studies suggested that the Day 90 visit for one intradermal injection could be replaced with two intradermal injections on Day 28 [14–16]. This updated Thai Red Cross (UTRC) regimen (2-2-2-0-2-0) was approved for use by the WHO expert consultation in 2006 [17]. Observational studies conducted at the Institut Pasteur du Cambodge (IPC) in Cambodia found that the fourth session of the UTRC regimen, administered on day 28, provides no additional benefit [18,19]. Based on the findings from Cambodia and other studies with shorter regimen, WHO, in its 2018 position paper, recommended a shortened intradermal, 1-week, three-session IPC regimen (2-2-2-0-0-0). It is a 2-site intradermal regimen with a dose of 0.1 mL per site, administered on Days 0, 3, and 7 [20]. However, many Asian and African countries where majority (nearly 99%) of human rabies deaths occur [1], still use UTRC or intramuscular regimen [21,22] (S1 Appendix).

In India, the National Rabies Control Programme (NRCP) currently recommends the UTRC regimen [23]. Purified Chick Embryo Cell vaccine (PCECV) and the Purified Vero Cell Rabies vaccine (PVRV) are currently used in India. A recent nationwide survey indicated that only 40% complete the prescribed UTRC or intramuscular Essen regimen (0.5 ml, five doses on days 0, 3, 7, 14, and 28) [2]. Additionally, 33% of rabies vaccinations are still administered through the intramuscular route, and in nearly 20% of the health facilities reported nonavailability of anti-rabies vaccines [24]. Introduction of abridged regimens such as IPC can increase compliance and coverage and ensure vaccine availability in all health facilities. To support policy decisions, evidence from immunogenicity studies is required. In this context, we conducted a randomised vaccine trial among healthy volunteers to evaluate the immunogenicity and safety of IPC regimen compared with the UTRC regimen, using both the PCECV and the PVRV. The trial was designed as an equivalence study because the main policy question was whether the 1-week IPC regimen could be considered immunologically comparable to the currently recommended 1-month UTRC regimen and therefore potentially replace it in programme settings. Earlier studies have shown that RVNA titres were similar on Day 28 and Day 42 [18,25].

## Methods

### Study design and participants

This phase 4, open-label, equivalence, randomised, active-controlled, nonregulatory trial was conducted at the clinical trial centre at Mahatma Gandhi Medical College and Research Institute, Sri Balaji Vidyapeeth, in Pondicherry, India. The protocol was approved by the Institutional Human Ethics Committee of Indian Council of Medical Research National Institute of Epidemiology (Approval no: NIE/IHEC/202110-02) and the ethics committee of the Mahatma Gandhi Medical College and Research Institute (Approval no: MGMCRI/2022/(07)/IHEC/38). The study complied with the ethical standards outlined in the National ethical guidelines for biomedical and health research involving human participants. The trial was prospectively registered with the Clinical Trial Registry – India (CTRI/2023/10/058418), URL: https://ctri.nic.in/Clinicaltrials/pmaindet2.php?EncHid=OTAxMDA=&Enc=&userName=) The study was initially planned at two sites. Since the approvals did not come in time and there was urgency to complete the recruitment from the funding agency, the trial was conducted only at one site. Patients or the public did not contribute to the design, conduct or reporting of the trial. This study is reported in accordance with the CONSORT 2025 statement and reporting of noninferiority and equivalence randomised trial; Extension of CONSORT 2010 statement (S1 Checklist).

Written informed consent was obtained from all the participants prior to enrolment in either English or local Tamil language. Participants were compensated for their travel and other incidental costs. Participants were clinically screened for eligibility before inclusion in the study. Eligible participants were healthy adult volunteers aged between 18 and 45 years. Individuals were excluded if they had a history of animal bite or anti-rabies vaccination in the past, were immunocompromised or receiving immunosuppressive therapy, had a history of allergy to any vaccine, or were receiving concomitant medications such as anti-malarial drugs, or corticosteroids that could potentially interfere with immune response.

### Randomisation and masking

Eligible participants were randomly assigned to one of the four study groups receiving either the IPC or UTRC regimen with either PCECV or the PVRV in a 1:1:1:1 allocation ratio. Randomisation was performed using permuted blocks of varying sizes (4, 8, and 12) to ensure balance among the four groups. The allocation sequences were generated prior to the study initiation using the *ralloc* package in STATA SE v17.0 software. We used both PCECV and PVRV for the trial since both types of vaccines are currently used in public health facilities of India for PEP.

Randomisation was implemented by the site staff, with allocation concealment ensured through an in-house secured web-based online application that administered intervention assignments. Participant enrolment was carried out by the site staff, and data collection and management were done using the RedCap software v14.0. Although participants and study staff were aware of the interventions assigned, laboratory personnel responsible for testing immunogenicity remained blinded to the allocated interventions.

### Procedures

The first participant was recruited on 10 October 2023, and the last participant was recruited on 23 December 2023. The last 365-day sample was collected on 24 December 2024. PCECV and PVRV were used for the study to administer either the IPC or the UTRC regimen. The UTRC regimen (2-2-2-0-2-0) was administered intradermally in both deltoids, with a dose of 0.1 mL to each deltoid region on Days 0, 3, 7, and 28. Similarly, the IPC regimen (2-2-2-0-0-0) was administered intradermally in both deltoids, with a dose of 0.1 mL to each deltoid region on Days 0, 3, and 7. Individuals randomised in Arm 1 received the PCECV-IPC regimen, whereas those in Arm 2 received the PCECV-UTRC regimen. Likewise, individuals randomised in Arm 3 received the PVRV-IPC regimen, and participants of Arm 4 received the PVRV-UTRC regimen. PCECV (ChiroRab, Chiron Behring, Batch No.4211) was supplied as lyophilised powder containing inactivated rabies virus (Flury LEP strain) with a potency of ≥ 2.5 IU. Similarly, PVRV (Rabivax, Serum Institute of India, Batch No. 1483M035), containing inactivated purified rabies antigen derived from Pitman-Moore Strain 3218 was supplied as lyophilised powder, with a potency of ≥ 2.5 IU. A 1.0 mL sterile water ampoule was supplied along with the vaccine vial for reconstitution. The vaccines were transported and stored at a temperature of 2–8 °C until use. The reconstituted vaccine was administered within 4 hours, and any remaining vaccine was discarded. In between administration, the vial was kept in the cold chain.

The participants visited the site on days 0, 3, 7, 14, 28, 42, 90, 180, and 365. About 3–5 mL of blood was drawn from each participant on days 0, 14, 28, 42, 90, 180, and 365. The blood samples were centrifuged, and the serum was separated on the same day of blood sample collection. The serum was stored at −80 °C and transported under frozen condition until testing. Serum was tested for rabies virus neutralising antibodies (RVNA) using the Rapid Fluorescent Focus Inhibition Test (RFFIT) by standard protocol at the ICMR National Institute of Virology, Pune, India. As part of quality assurance, 100 randomly selected serum samples were tested at the National Institute of Mental Health and Neurosciences (NIMHANS), Bangalore, India, a WHO Collaborating Centre for Reference and Research in Rabies.

The participants were monitored closely for 30 min following each dose of vaccine for potential post-vaccination reactogenicity. Safety evaluations for adverse events were conducted during follow-up site visits. Additionally, participants were provided with a diary card to record any adverse events during 14-day period following each vaccine. Furthermore, the participants were telephonically followed up on days 35, 60, and every month for 1 year.

Participants provided information about any adverse events (AEs) reported during the follow-up visits or telephonic calls. Participants were encouraged to visit the site at any time or call the site team to report any AEs. AEs were monitored closely on a daily basis till resolution. AEs were graded as mild, moderate and severe based on the Division of AIDS (DAIDS) Table for Grading the Severity of Adult and Pediatric Adverse Events, Version 2.1 [26]. The participants were also monitored for any serious adverse events (SAEs) throughout the study period. The causal relation between the SAEs and study products was evaluated by the site investigators using the WHO-UMC system. Adverse events occurring within 7 days post-administration of the vaccine were considered solicited AEs, while all other adverse events were considered unsolicited. The site investigators used their clinical judgement to identify the causal relation between the AEs and study products, considering the nature of the reaction, temporality and biological plausibility. There were no crossover events during the trial. A Data and Safety Monitoring Board (DSMB) was constituted to monitor participant safety, study conduct, and data quality.

#### Estimation of rabies virus neutralising antibody titres.

All procedures were performed inside a validated Class II Type A2 biological safety cabinet. A standard RFFIT (WHO, 1996) was performed using Baby Hamster Kidney (BHK-21) (ATCC CCL-10) cell line and Challenge Virus Standard 11 (CVS-11) strain of rabies virus, with minor modifications. Briefly, serum samples were heat-inactivated at 56 °C and two-fold dilutions were prepared in Dulbecco’s Modified Eagle Medium (DMEM), in 96-well microtiter plates (Corning). Subsequently, 100 μL of CVS-11 virus (containing 100 Fluorescent Focus Forming Dose_50_, FFD_50_) was added to diluted samples, and the test plates incubated at 37 °C for 1 hour. Mock-infected cells and virus controls were included. The Third WHO International Standard for Anti-Rabies Immunoglobulins (NIBSC Code 19/244, UK), diluted to 2 IU/mL was included as a reference standard. Subsequently, 100 μL of a suspension of BHK-21 cells (containing 5 x 10^4^ cells) was added to the test wells, and the plates incubated for 40 hours in a 37 °C incubator under 5% CO_2_. Subsequently, the cell monolayers were fixed using cold acetone, and residual virus infectivity was determined by direct immunofluorescent staining using an anti-rabies nucleocapsid conjugate (BioRad, Cat. No. 3572114). The wells were counterstained using Evans Blue (0.0002%) for 1 min, and the plates were examined using a FLoid Cell Imaging Station (Thermo Scientific). Sample dilutions showing 50% neutralisation of the challenge virus were documented, and the corresponding antibody titres (IU/mL) were reported in correlation with those yielded by the reference serum. The lowest level of detection was <0.125 IU/mL, and the highest level of detection was 256 IU/mL. When the detection level was <0.125 IU/mL, we used 0.125 IU/mL to calculate the GMCs.

### Outcomes

The primary outcome was Day 42 geometric mean concentration ratio (GMR) of RVNA for the IPC versus UTRC regimen for each vaccine type. The secondary outcome was the proportion of participants with RVNA titres ≥0.5 IU/mL on day 14, 28, 42, 90, 180, and 365. Safety outcomes included the proportion of participants with solicited and unsolicited AEs and SAEs.

### Statistical analysis

A sample size of 400 participants was calculated, with 100 participants in each of the four study arms (PCECV-IPC, PCECV-UTRC, PVRV-IPC, and PVRV-UTRC). The equivalence framework was prespecified to determine whether the shorter regimen produced antibody responses sufficiently comparable to the standard regimen to support immunological comparability. The sample size was calculated to demonstrate equivalence, assuming the GMR as 0.9, equivalence margin of 0.72–1.04 (which constitutes 20% for the lower margin and 15% for the upper margin), alpha of 0.025, power of 90%, a coefficient of variation as 28% and allowed for 20% loss to follow-up. As rabies is almost invariably fatal once clinical symptoms appear, a narrower equivalence margin was considered appropriate. Accordingly, a conservative equivalence margin of 0.72–1.04 for the Day 42 GMR was selected instead of the conventional margin of 0.67–1.50 commonly used in equivalence studies of vaccines against nonfatal diseases [27]. This conservative margin was chosen to increase sensitivity for detecting meaningful differences in peak antibody responses during the incubation period, thereby strengthening confidence in immunological comparability of the two regimens.

We used the modified intention-to-treat (mITT) population, rather than an intention-to-treat analysis, for the primary outcome because the Day 42 GMC/GMR is a laboratory-based endpoint that required an evaluable blood sample. The primary analysis used the mITT analysis set, which included study participants who received at least one dose of the vaccine as per the randomised regimen allocation and had a day 42 blood sample available within or outside the specified window period of ±2 days. All study participants who received the complete PEP regimen in the respective randomised group and adhered to the protocol-specified visits were considered for the per-protocol analysis. The safety analysis set included all participants who had received at least one dose of vaccine in their respective arms.

All analyses were performed using Stata version 17.1. Continuous variables were summarised as mean with standard deviation and median with interquartile range. Categorical variables were summarised as frequencies and percentages. GMCs were calculated as the exponentiation of arithmetic mean of the natural log-transformed RVNA titres measured in IU/mL for days 0, 14, 28, 42, 90, 180, and 365. The log-transformed RVNA titres (IU/mL) were compared between the study arms (PCECV-IPC and PCECV-UTRC; PVRV-IPC, and PVRV-UTRC) using an independent sample *t*-test. The GMR (GMC of PCECV-IPC to GMC PCECV-UTRC and GMC of PVRV-IPC to GMC of PVRV-UTRC) was calculated as the exponentiation of the mean difference and 95% CI for the GMR was calculated using the exponentiation of lower and upper confidence interval for mean difference. The equivalence between the two regimens was concluded if the 95% confidence limits of the observed GMR on day 42 were within the range between 0.72 and 1.04.

Among the individuals seronegative at baseline, serum RVNA concentration of ≥0.5 IU/mL after vaccination was considered as evidence of an adequate immune response after vaccination for participants [7]. Participants with RVNA titres <0.5 IU/mL, including those with detectable titres below this threshold, were considered seronegative. The proportion of participants with RVNA concentration ≥0.5 IU/mL, was calculated, along with 95% CI, for days 0, 14, 28, 42, 90, 180, and 365 and compared between the regimens for two different vaccines (PCECV-IPC and PCECV-UTRC, PVRV-IPC and PVRV-UTRC) using the chi-square test. We did not anticipate seropositivity at baseline, since one of the trial’s exclusion criteria was receipt of rabies vaccination in the past and a history of dog bites. We did a stratified analysis for seropositive and seronegative individuals at baseline as an additional analysis. OpenAI (ChatGPT (GPT 5.6 Luna) was used to check grammar and improve writing style for clarity.

## Results

A total of 418 participants were screened for eligibility, of whom 400 were randomly assigned to four study arms (100 each to PCECV-IPC, PCECV-UTRC, PVRV-IPC, and PVRV-UTRC). All 400 participants received at least one vaccine dose, and their baseline blood sample was collected. Overall, 362 (90.5%) completed the PEP regimen and blood draw on Day 42, with 92 in the PCECV-IPC, 88 in the PCECV-UTRC, 92 in the PVRV-IPC, and 90 in the PVRV-UTRC (Fig 1).

**Fig 1:**
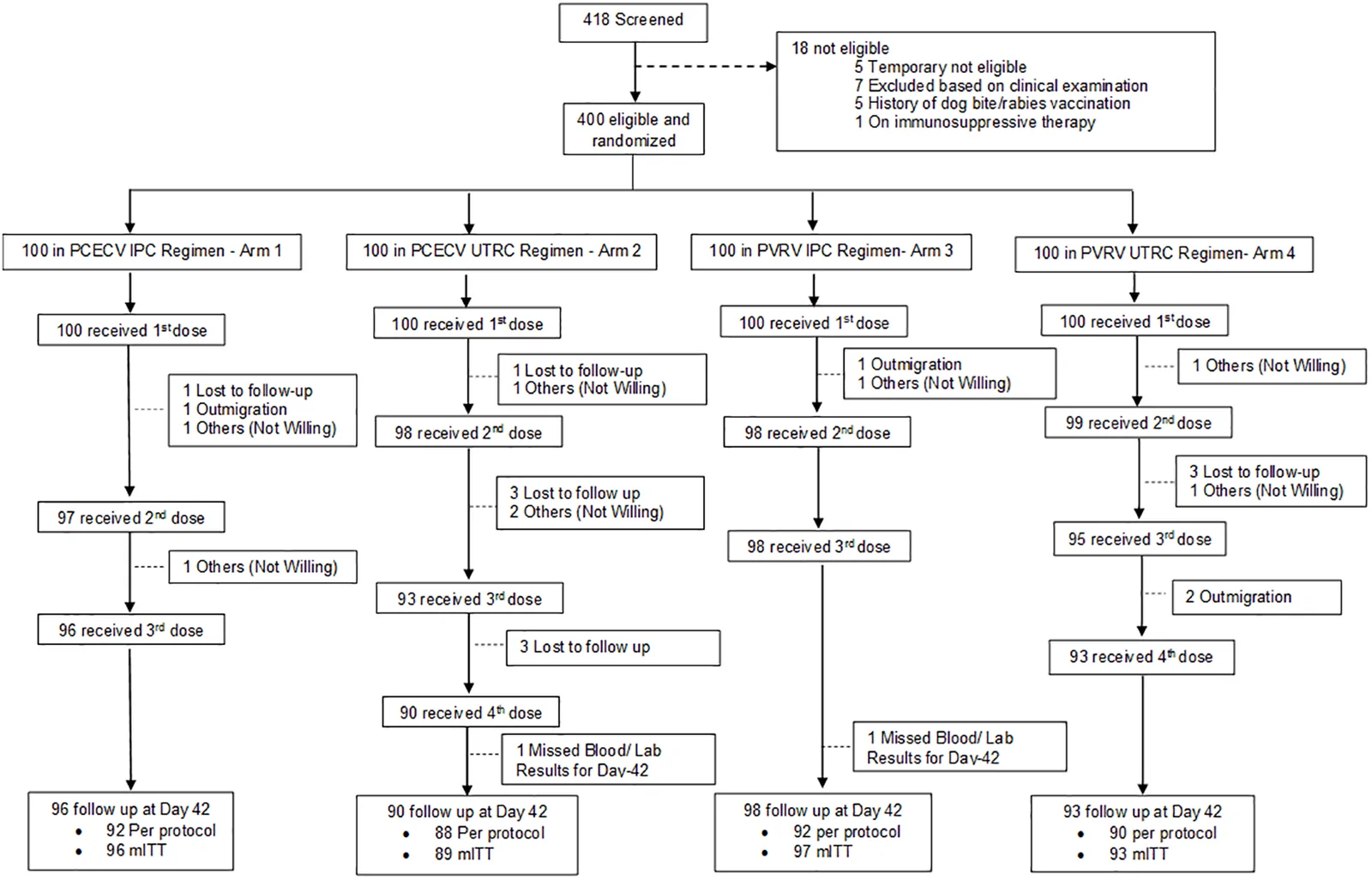
Study flow diagram.

The baseline demographic and anthropometric characteristics of the study participants were balanced across four study arms (Table 1). Most participants were male, with proportions ranging from 70% to 79% across the arms. The mean age of the participants was similar between the groups, ranging from 20.6 to 21.2 years. The height and weight of the participants were similar across the four groups. (Table 1)

**Table 1:** Baseline characteristics of study participants by vaccine arms.

Category	Arm 1	Arm 2	Arm 3	Arm 4	Total
	*N* = 100	*N* = 100	*N* = 100	*N* = 100	*N* = 400
**Sex**	*N* (%)	*N* (%)	*N* (%)	*N* (%)	*N* (%)
**Male**	78 (78.0)	74 (74.0)	79 (79.0)	70 (70.0)	301 (75.2)
**Female**	22 (22.0)	26 (26.0)	21 (21.0)	30 (30.0)	99 (24.7)
Age (Yrs)*	20.8 (3.3)	21.2 (4.4)	21.1 (3.6)	20.6 (2.6)	20.9 (3.6)
Height (cm)*	166.0 (8.0)	165.2 (8.1)	166.5 (8.3)	167.2 (9.0)	166.2 (8.4)
Weight (kg)*	60.6 (13.8)	62.1 (13.9)	63.9 (15.2)	64.4 (13.9)	62.8 (14.2)

Abbreviations: Arm1, PCECV-IPC Regimen (Day 0, 3, 7); Arm 2, PCECV-UTRC Regimen (Day 0, 3, 7, 28); Arm 3, PVRV-IPC Regimen (Day 0, 3, 7); Arm 4, PVRV-UTRC Regimen (Day 0, 3, 7, 28); PCECV, Purified Chick Embryo Cell rabies vaccine; PVRV, Purified Vero cell Rabies vaccine; IPC, Institut Pasteur du Cambodge; UTRC, Updated Thai Red Cross; N, Number of participants; *, Mean (SD).

On day 42, GMR of RVNA between the IPC and UTRC regimens in the mITT analysis was 0.59 (95% CI: 0.47, 0.75) for the PCECV and 0.76 (95% CI: 0.61, 0.95) for the PVRV. In the per-protocol analysis, the corresponding GMRs were 0.61 (95% CI: 0.48, 0.78) for the PCECV and 0.77 (95% CI: 0.61, 0.97) for the PVRV. Among participants who were seronegative at baseline (RVNA titres <0.5 IU/ml), the mITT analysis showed lower day 42 GMCs in the IPC regimen compared with the UTRC regimen, with GMR of 0.55 (95% CI, 0.43, 0.70) for PCECV and 0.76 (95% CI, 0.61, 0.95) for PVRV (Fig 2, Tables 2 and 3). Similar findings were observed after pooling the participants who received PCECV and PVRV (S2 Appendix). The baseline GMCs were comparable between the IPC and UTRC regimens for both vaccine groups (Tables 2 and 3).

**Table 2:** Geometric Mean Concentrations of rabies virus neutralising antibody titres in the PCECV-IPC and PCECV-UTRC regimen (Per Protocol & modified Intention to Treat (mITT)).

Blood Sample collection at Scheduled Visits	PCECV-IPC Regimen (Day 0, 3, 7)	PCECV-UTRC Regimen (Day 0, 3, 7, 28)		PCECV-IPC Regimen (Day 0, 3, 7)	PCECV-UTRC Regimen (Day 0, 3, 7, 28)	
Day	Per Protocol Analysis			mITT Analysis		
	Geometric Mean (GSD)	Geometric Mean (GSD)	Geometric Mean Ratio (GMR) 95% CI	Geometric Mean (GSD)	Geometric Mean (GSD)	Geometric Mean Ratio (GMR) 95% CI
	***n* = 92**	***n* = 88**		***n* = 96**	***n* = 89**	
Day 0	0.34 (6.83)	0.32 (6.25)	1.08 (0.62, 1.88)	0.34 (6.72)	0.31 (6.20)	1.09 (0.63, 1.87)
Day 14	15.76 (2.71)	17.18 (2.35)	0.92 (0.70, 1.21)	15.43 (2.70)	17.16 (2.34)	0.90 (0.69, 1.18)
Day 28	15.18 (2.54)	14.33 (2.32)	1.06 (0.82, 1.38)	14.66 (2.56)	14.13 (2.33)	1.04(0.80, 1.35)
Day 42	12.86 (2.41)	20.91 (2.09)	0.61 (0.48, 0.78)	12.25 (2.45)	20.69 (2.10)	0.59 (0.47, 0.75)
Day 90	7.20 (2.92)	17.59 (2.47)	0.41 (0.31, 0.55)	6.92 (2.97)	17.43 (2.47)	0.40 (0.30, 0.53)
Day 180	5.29 (2.67)	6.73 (2.55)	0.79 (0.59, 1.04)	5.30 (2.65)	6.79 (2.55)	0.78 (0.59, 1.03)
Day 365	6.29 (2.73)	6.88 (2.69)	0.91 (0.68, 1.23)	6.21 (2.69)	6.94 (2.68)	0.90 (0.67, 1.19)
**Seronegative at baseline**	***n* = 71**	***n* = 69**		***n* = 74**	***n* = 70**	
Day 0	–	–	–	–	–	–
Day 14	12.17 (2.34)	13.09 (1.98)	0.93 (0.72, 1.20)	12.15 (2.31)	13.12 (1.97)	0.93 (0.72, 1.19)
Day 28	11.26 (2.21)	10.71 (1.91)	1.05 (0.83, 1.34)	11.05 (2.21)	10.56 (1.92)	1.05 (0.82, 1.33)
Day 42	10.21 (2.20)	18.23 (2.03)	0.56 (0.44, 0.72)	9.83 (2.23)	18.02 (2.03)	0.55 (0.43, 0.70)
Day 90	5.31 (2.69)	14.33 (2.41)	0.37 (0.27, 0.51)	5.06 (2.72)	14.21 (2.40)	0.36 (0.26, 0.49)
Day 180	3.70 (1.99)	4.94 (1.97)	0.75 (0.60, 0.94)	3.71 (1.98)	5.02 (1.98)	0.74 (0.59, 0.93)
Day 365	4.45 (2.11)	5.21 (2.35)	0.85 (0.65, 1.12)	4.48 (2.10)	5.30 (2.36)	0.84 (0.65, 1.10)
**Seropositive at baseline**	***n* = 21**	***n* = 19**		***n* = 22**	***n* = 19**	
Day 0	10.42 (2.63)	9.26 (2.56)	1.13 (0.61, 2.08)	9.97 (2.63)	9.26 (2.56)	1.08 (0.59, 1.97)
Day 14	37.74 (2.66)	46.09 (1.95)	0.82 (0.48, 1.41)	34.08 (2.91)	46.09 (1.95)	0.74 (0.42, 1.31)
Day 28	41.67 (1.82)	41.31 (1.78)	1.01 (0.69, 1.47)	37.46 (2.16)	41.31 (1.78)	0.91 (0.59, 1.40)
Day 42	28.04 (2.04)	34.42 (1.91)	0.81 (0.53, 1.26)	25.67 (2.25)	34.42 (1.91)	0.75 (0.47, 1.19)
Day 90	20.16 (1.81)	37.03 (1.73)	0.54 (0.38, 0.79)	19.95 (1.79)	37.03 (1.73)	0.54 (0.38, 0.77)
Day 180	17.67 (2.36)	20.66 (2.46)	0.86 (0.49, 1.50)	17.59 (2.31)	20.66 (2.46)	0.85 (0.49, 1.47)
Day 365	20.16 (2.43)	18.51 (2.20)	1.09 (0.64, 1.87)	18.73 (2.54)	18.51 (2.20)	1.01 (0.58, 1.75)

Abbreviations: PCECV, Purified Chick Embryo Cell rabies vaccine; PVRV, Purified Vero cell Rabies vaccine; IPC, Institut Pasteur du Cambodge; UTRC, Updated Thai Red Cross; mITT, Modified Intention to treat; GSD, Geometric standard deviation.

**Table 3:** Geometric Mean Concentrations rabies virus neutralising antibody titres in the PVRV-IPC and PVRV-UTRC Regimen (Per Protocol & modified Intention to Treat (mITT)).

Blood sample collection at scheduled visits	PVRV-IPC Regimen	PVRV-UTRC Regimen		PVRV-IPC Regimen	PVRV-UTRC Regimen	
	Per protocol analysis			mITT analysis		
Day	Geometric Mean (GSD)	Geometric Mean (GSD)	Geometric Mean Ratio (GMR) 95% CI	Geometric Mean (GSD)	Geometric Mean (GSD)	Geometric Mean Ratio (GMR) 95% CI
	***n* = 92**	***n* = 90**		***n* = 97**	***n* = 93**	
Day 0	0.37 (7.71)	0.39 (7.81)	0.95 (0.52, 1.72)	0.37 (7.79)	0.40 (8.00)	0.93 (0.51, 1.67)
Day 14	16.37 (2.50)	16.63 (2.45)	0.98 (0.76, 1.28)	16.72 (2.50)	16.49 (2.43)	1.01 (0.78, 1.32)
Day 28	17.12 (2.22)	16.00 (2.31)	1.07 (0.84, 1.36)	17.18 (2.19)	16.00 (2.34)	1.07 (0.85, 1.36)
Day 42	14.62 (2.25)	18.95 (2.09)	0.77 (0.61, 0.97)	14.48 (2.23)	18.99 (2.10)	0.76 (0.61, 0.95)
Day 90	7.53 (2.98)	15.63 (2.55)	0.48 (0.36, 0.65)	7.44 (2.95)	15.64 (2.53)	0.48 (0.36, 0.64)
Day 180	5.29 (2.82)	6.86 (2.57)	0.77 (0.58, 1.03)	5.17 (2.81)	6.89 (2.55)	0.75 (0.57, 1.00)
Day 365	5.79 (2.65)	7.58 (2.28)	0.76 (0.59, 1.00)	5.76 (2.62)	7.54 (2.29)	0.76 (0.59, 0.99)
**Seronegative at baseline**	***n* = 71**	***n* = 68**		***n* = 75**	***n* = 70**	
Day 0	–	–	–	–	–	–
Day 14	11.71 (1.98)	12.79 (2.18)	0.92 (0.72, 1.17)	11.92 (1.98)	12.74 (2.16)	0.94 (0.74, 1.19)
Day 28	12.78 (1.81)	11.91 (1.95)	1.07 (0.87, 1.33)	12.94 (1.81)	11.77 (1.96)	1.10 (0.89, 1.35)
Day 42	11.94 (1.99)	15.68 (1.95)	0.76 (0.61, 0.96)	11.79 (1.97)	15.53 (1.94)	0.76 (0.61, 0.95)
Day 90	5.52 (2.70)	12.57 (2.47)	0.44 (0.32, 0.61)	5.45 (2.67)	12.53 (2.45)	0.43 (0.32, 0.59)
Day 180	3.52 (2.08)	5.21 (2.35)	0.68 (0.52, 0.88)	3.45 (2.06)	5.23 (2.33)	0.66 (0.51, 0.85)
Day 365	4.28 (2.16)	5.89 (2.02)	0.73 (0.57, 0.93)	4.27 (2.13)	5.83 (2.02)	0.73 (0.58, 0.93)
**Seropositive at baseline**	***n* = 21**	***n* = 22**		***n* = 22**	***n* = 23**	
Day 0	14.98 (1.92)	13.67 (1.96)	1.10 (0.73, 1.65)	15.50 (1.93)	14.18 (1.98)	1.09 (0.73, 1.64)
Day 14	50.80 (1.95)	37.46 (2.09)	1.36 (0.88, 2.09)	51.33 (1.93)	37.46 (2.09)	1.37 (0.90, 2.10)
Day 28	46.01 (1.76)	39.90 (1.86)	1.15 (0.80, 1.66)	45.25 (1.74)	40.72 (1.85)	1.11 (0.78, 1.58)
Day 42	28.98 (2.29)	34.08 (1.89)	0.85 (0.54, 1.34)	29.11 (2.25)	35.03 (1.89)	0.83 (0.54, 1.29)
Day 90	21.54 (1.97)	30.05 (2.03)	0.72 (0.47, 1.10)	21.24 (1.94)	30.13 (1.99)	0.71 (0.47, 1.06)
Day 180	20.83 (1.96)	16.00 (1.97)	1.30 (0.86, 1.97)	20.59 (1.94)	16.00 (1.94)	1.29 (0.86, 1.91)
Day 365	17.85 (2.32)	16.51 (2.00)	1.08 (0.66, 1.77)	17.75 (2.27)	16.49 (1.97)	1.08 (0.67, 1.72)

Abbreviations: PCECV, Purified Chick Embryo Cell rabies vaccine; PVRV, Purified Vero cell Rabies vaccine; IPC, Institut Pasteur du Cambodge; UTRC, Updated Thai Red Cross; mITT, Modified Intention to treat; GSD, Geometric standard deviation.

**Fig 2:**
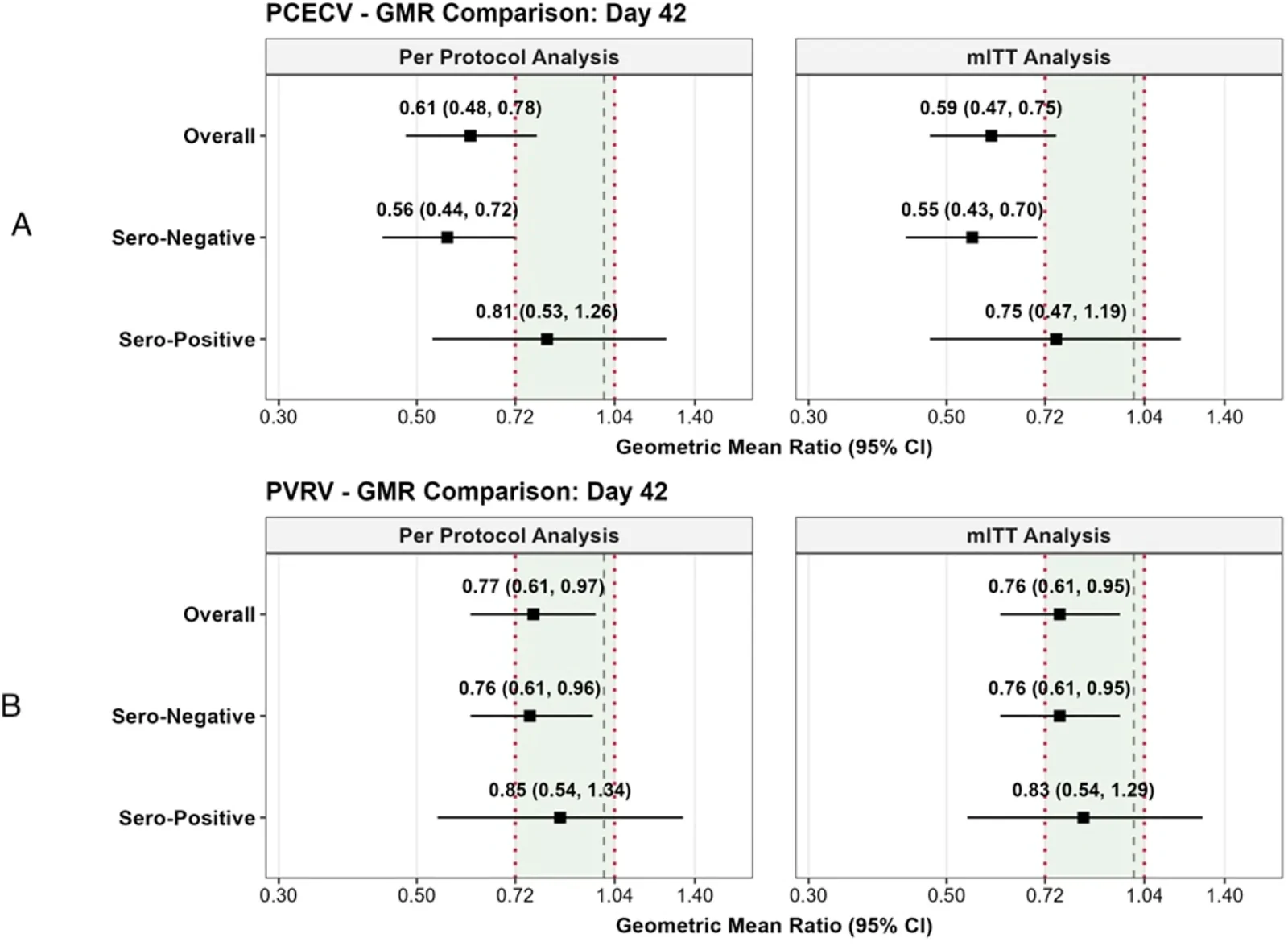
Geometric mean concentration ratio of rabies virus neutralising antibody titres in the (a) PCECV-IPC regimen and PCECV-UTRC regimen (b) PVRV-IPC Regimen and PVRV-UTRC regimen.

At baseline, 22.8% (21/92) participants in the PCECV-IPC arm, 21.6% (19/88) in the PCECV-UTRC arm, 22.8% (21/92) in the PVRV-IPC arm, and 24.4% (22/90) in the PVRV-UTRC arm had RVNA titre of ≥0.5 IU/mL (Table 4) in the per-protcol population. On day 42, 100% of the participants seronegative at baseline had evidence of adequate immune response (RVNA titre of ≥0.5 IU/mL) across all four arms for both modified ITT and per-protocol analysis (Table 4).

**Table 4:** Proportion of Individuals with Rabies virus neutralising antibody titres ≥0.5 IU/mL in the PCECV-IPC, PCECV-UTRC, PVRV-IPC and PVRV-UTRC regimens.

Days	Per Protocol Analysis		mITT analysis	
	*n/N* (%)	*n/N* (%)	*n/N* (%)	*n/N* (%)
	**PCECV-IPC (*n* = 92)**	**PCECV-UTRC (*n* = 88)**	**PCECV-IPC (*n* = 96)**	**PCECV-UTRC (*n* = 89)**
Day 0	21/92 (22.8)	19/88 (21.6)	22/96 (22.9)	19/89 (21.3)
Day 14	92/92 (100.0)	88/88 (100.0)	95/95 (100.0)	89/89 (100.0)
Day 28	92/92 (100.0)	88/88 (100.0)	95/95 (100.0)	89/89 (100.0)
Day 42	92/92 (100.0)	88/88 (100.0)	96/96 (100.0)	89/89 (100.0)
Day 90	92/92 (100.0)	88/88 (100.0)	96/96 (100.0)	89/89 (100.0)
Day 180	92/92 (100.0)	88/88 (100.0)	96/96 (100.0)	89/89 (100.0)
Day 365	92/92 (100.0)	87/87 (100.0)	96/96 (100.0)	88/88 (100.0)
	**PVRV-IPC (*n* = 92)**	**PVRV-UTRC (*n* = 90)**	**PVRV-IPC (*n* = 97)**	**PVRV-UTRC (*n* = 93)**
Day 0	21/92 (22.8)	22/90 (24.4)	22/97 (22.7)	23/93 (24.7)
Day 14	92/92 (100.0)	90/90 (100.0)	95/95 (100.0)	92/92 (100.0)
Day 28	92/92 (100.0)	90/90 (100.0)	97/97 (100.0)	93/93 (100.0)
Day 42	92/92 (100.0)	90/90 (100.0)	97/97 (100.0)	93/93 (100.0)
Day 90	92/92 (100.0)	88/88 (100.0)	96/96 (100.0)	91/91 (100.0)
Day 180	92/92 (100.0)	90/90 (100.0)	97/97 (100.0)	93/93 (100.0)
Day 365	90/90 (100.0)	90/90 (100.0)	95/95 (100.0)	93/93 (100.0)

Abbreviations: PCECV, Purified Chick Embryo Cell rabies vaccine; PVRV, Purified Vero cell Rabies vaccine; IPC, Institut Pasteur du Cambodge; UTRC, Updated Thai Red Cross; ITT, Intention to treat.

GMCs between the IPC and UTRC regimens were comparable across days 14, 28, 180, and 365 for both the PCECV and PVRV groups. This trend remained unchanged between the per-protocol and mITT analyses. However, on day 90, GMCs were higher in the UTRC regimen than in the IPC regimen (PCECV: 17.59 IU/mL vs. 7.20 IU/mL and PVRV: 15.63 IU/mL vs. 7.53 IU/mL) (Tables 2 and 3). This pattern was consistent among participants who were seronegative and seropositive at baseline. Across all four study arms, all participants, including seropositive and seronegative at baseline, had an RVNA level of ≥0.5 IU/mL on days 14, 28, 90, 180, and 365, irrespective of the analysis population (Table 4).

A total of 71 solicited adverse events were noted among 33 of the 100 participants in the PCECV-IPC arm, compared with 64 solicited adverse events among 31 of the 100 participants in the PCECV-UTRC arm. In the PVRV group, 38 solicited adverse events were noted among 22 of the 100 participants in the IPC arm, and 59 solicited adverse events were noted among 26 of the 100 participants in the UTRC arm. Most solicited adverse events were mild to moderate in intensity and resolved without sequelae. Most commonly reported local solicited adverse events were redness, pruritus, swelling, and tenderness. Common systemic symptoms included fever, fatigue, headache, extreme tiredness, joint pain (Table 5 and 6). One death due to a road traffic accident, 9 months after the last dose of vaccination, was reported and was unrelated to the study product.

**Table 5:** Distribution of solicited and unsolicited adverse events after vaccination for PCECV.

	PCECV					
	IPC Regimen (*n* = 100)			UTRC Regimen (*n* = 100)		
	Number of participants	Percentage (95% CI)	Number of events	Number of participants	Percentage (95% CI)	Number of events
**Any solicited events**	33	33 (23.9,43.1)	71	31	31 (22.1, 41.0)	64
**Local adverse events**						
Redness	17	17 (10.2,25.9)	23	15	15 (8.6, 23.5)	30
Pruritus	11	11 (5.6, 18.8)	11	10	10 (4.9, 17.6)	11
Swelling	9	9 (4.2, 16.4)	11	7	7 (2.9, 13.9)	7
Pain/tenderness	5	5 (1.6,11.2)	5	5	5 (1.6, 11.2)	5
**Systemic adverse events**						
Fever	6	6 (2.2, 12.6)	6	–	–	–
Fatigue	5	5 (1.6, 11.2)	5	3	3 (0.6, 8.5)	3
Headache	4	4 (1.1, 9.9)	4	2	2 (0.2, 7.0)	2
Extreme tiredness	2	2 (0.2, 7.0)	2	–	–	–
Chills	1	1 (0.0, 5.4)	1	–	–	–
Nausea	1	1 (0.0, 5.4)	1	–	–	–
Abdominal pain	1	1 (0.0, 5.4)	1	–	–	–
Myalgia	1	1 (0.0, 5.4)	1	2	2 (0.2, 7.0)	2
Joint pain	–	–	–	3	3 (0.6, 8.5)	4
**Any unsolicited events**	22	22 (14.3, 31.4)	26	18	18 (11.0, 26.9)	19
Upper respiratory tract infection	14	14 (7.9, 22.4)	16	9	9 (4.2, 16.4)	10
Cough	3	3 (0.6, 8.5)	4	2	2 (0.2, 7.0)	2
Runny nose	1	1 (0.0, 5.4)	1	3	3 (0.6, 8.5)	3
Fever	1	1 (0.0, 5.4)	1	2	2 (0.2, 7.0)	2
Headache	1	1 (0.0, 5.4)	1	–	–	–
Itching	1	1 (0.0, 5.4)	1	–	–	–
Rash	1	1 (0.0, 5.4)	1	–	–	–
Acute gastroenteritis	1	1 (0.0, 5.4)	1	–	–	–
Abdominal pain	–	–	–	1	1 (0.0, 5.4)	1
Nasal congestion	–	–	–	1	1 (0.0, 5.4)	1

Abbreviations: PCECV, Purified Chick Embryo Cell rabies vaccine; IPC, Institut Pasteur du Cambodge; UTRC; Updated Thai Red Cross.

**Table 6:** Distribution of solicited and unsolicited adverse events after vaccination for PVRV.

	PVRV					
	IPC Regimen Day (0, 3, 7) (*n* = 100)			UTRC Regimen Day (0, 3, 7, 28) (*n* = 100)		
	Number of participants	Percentage (95% CI)	Number of events	Number of participants	Percentage (95% CI)	Number of events
**Any solicited events**	22	22 (14.3, 31.4)	38	26	26 (17.7, 35.7)	59
**Local adverse events**						
Swelling	6	6 (2.2, 12.6)	9	9	9 (4.2, 16.4)	12
Pruritus	6	6 (2.2, 12.6)	6	7	7 (2.9, 13.9)	9
Redness	5	5 (1.6, 11.2)	5	11	11 (5.6, 18.8)	17
Pain/tenderness	2	2 (0.2, 7.0)	2	5	5 (1.6, 11.2)	5
**Systemic adverse events**						
Fever	6	6 (2.2, 12.6)	6	4	4 (1.1, 9.9)	5
Headache	4	4 (1.1, 9.9)	4	2	2 (0.2, 7.0)	2
Fatigue	1	1 (0.0, 5.4)	1	3	3 (0.6, 8.5)	4
Chills	1	1 (0.0, 5.4)	1	–	–	–
Myalgia	1	1 (0.0, 5.4)	1	1	1 (0.0, 5.4)	2
Nausea	1	1 (0.0, 5.4)	1	–	–	–
Joint pain	1	1 (0.0, 5.4)	1	1	1 (0.0, 5.4)	1
Extreme tiredness	1	1 (0.0, 5.4)	1	–	–	–
Lymph node swelling	–	–	–	1	1 (0.0, 5.4)	1
Faintness/dizziness	–	–	–	1	1 (0.0, 5.4)	1
**Any unsolicited events**	20	20 (12.6, 29.1)	22	21	21 (13.5, 30.3)	25
Upper respiratory tract infection	10	10 (4.9, 17.6)	10	12	12 (6.3, 20.0)	14
Fever	3	3 (0.6, 8.5)	3	–	–	–
Itching	2	2 (0.2, 7.0)	3	3	3 (0.6, 8.5)	3
Vomiting	1	1 (0.0, 5.4)	1	–	–	–
Ear block	1	1 (0.0, 5.4)	1	–	–	–
Sore throat	1	1 (0.0, 5.4)	1	–	–	–
Cough	1	1 (0.0, 5.4)	1	2	2 (0.2, 7.0)	2
Nasal block	1	1 (0.0, 5.4)	1	–	–	–
Hair loss	1	1 (0.0, 5.4)	1			
Nasal congestion	–	–	–	2	2 (0.2, 7.0)	2
Lower back pain	–	–	–	1	1 (0.0, 5.4)	1
Eye infection	–	–	–	1	1 (0.0, 5.4)	1
Runny nose	–	–	–	2	2 (0.2, 7.0)	2

Abbreviations: PVRV, Purified Vero cell Rabies vaccine; IPC, Institut Pasteur du Cambodge; UTRC, Updated Thai Red Cross.

## Discussion

In this randomised, open-label trial among healthy volunteers, we compared the immunogenicity and safety of a shortened 1-week intradermal IPC regimen with the standard 1-month intradermal UTRC regimen using two widely used cell-culture rabies vaccines. The abridged IPC regimen did not meet the prespecified equivalence criteria in terms of the GMR at day 42. Day 42 endpoint was selected because it lies within the early post-exposure period during which vaccine-induced antibody responses are clinically important. The IPC and UTRC regimens are identical through day 7 and differ only with an additional dose in UTRC on Day 28. Therefore, assessment at Day 42 allowed comparison of immune responses after completion of both regimens and specifically captured the immunological effect of an additional Day 28 dose in UTRC regimen. All the participants who were seronegative at baseline achieved RVNA levels ≥0·5 IU/mL from day 14 onward and remained above this threshold through 1 year, indicating evidence of adequate immune response after vaccination [7]. In addition, RVNA levels remained ≥0.5 IU/mL for all the individuals who were seropositive at baseline. The incubation period for rabies is typically 2–3 months, but rarely could be as short as 1 week to as high as 1 year [28]. RVNA levels ≥0.5 IU/mL are therefore particularly critical during the early post-exposure period, generally considered to span from day 14 to day 90 after vaccination [7]. Our findings indicate that the abridged IPC regimen induced and maintained RVNA titres above the WHO recommended threshold from day 14 till 1 year.

The choice of the equivalence design for the trial was informed by the earlier study conducted by Cantaert *et al*. [18], which was the basis of WHO recommendation for IPC. In that study, the median rabies virus neutralising antibody concentration in the group of individuals bitten by rabies virus-positive dogs was 26·86 IU/mL (IQR: 22·68, 49·50) at Day 28, and 26·74 IU/mL (IQR: 11·78, 49·06) at Day 42 following the four dose UTRC regimen. Notably, in patients bitten by rabies virus-negative dogs, median titres were lower (26·86 IU/mL, IQR: 26·86, 54·50) at Day 42 compared to Day 28 titres (49·5 IU/mL, IQR: 26·86, 54·5)) after UTRC regimen. In another randomised trial, Bose *et al*. reported comparable GMC on Day 28 (27.93, 95% CI: 24.51, 31.82) and Day 42 (25.40, 95% CI: 21.98, 29.34) after UTRC regimen [25]. These findings suggest that after the initial immune response has been established, Day 28 UTRC dose may not produce a substantial further increase in RVNA concentrations. On this basis, we hypothesised that RVNA GMCs would be comparable between the IPC and UTRC regimens. We acknowledge, however, that a noninferiority design in terms of proportion of individuals having RVNA titre of ≥0.5 IU/mL would also have been reasonable. The present results should therefore be interpreted in relation to the prespecified equivalence hypothesis. The IPC regimen did not meet the prespecified Day 42 GMR equivalence criterion. However, all participants, irrespective of baseline serostatus and type of vaccine achieved RVNA titres ≥0.5 IU/mL from day 14 onward and maintained this response through 1 year.

The choice of Day 42 as the primary endpoint was intended to assess immunogenicity after completion of both vaccination schedules and within the early post-exposure period relevant to rabies prevention. However, this endpoint was not aligned by time since the last vaccine dose. In the IPC regimen, Day 42 occurred 35 days after the final day-7 dose, whereas in the UTRC regimen, Day 42 occurred 14 days after the additional day-28 dose. Thus, the higher GMCs in the UTRC group on Days 42 and 90 may reflect a transient booster response after the fourth dose rather than a clinically meaningful failure of the IPC regimen. Importantly, by day 180 and day 365, the RVNA GMCs were similar in both groups; however, the prespecified equivalence margin was not met for either of these timepoints. Higher GMCs among individuals at seropositive at baseline as compared to those who were seronegative indicates the prime boosting effect. Earlier studies have also reported such boosting among previously vaccinated individuals [29].

Few other studies conducted both outside and in India have evaluated shortened intradermal vaccination schedules. In addition to the immunogenicity study that supported the IPC schedule [18], Tarantola and his colleagues conducted a cohort analysis among patients with dog bites who were initiated on the Intradermal UTRC regimen and concluded that there was no significant difference in survival among patients with dog bites who received 3 versus 4 or more sessions [19]. Two Indian vaccine trial studies, one conducted among healthy volunteers and the other among patients with dog bites, reported RVNA titres ≥0.5 IU/mL on Day 14 in all participants after a 1-week, three-session regimen with four intradermal injections (4-4-4-0-0-0) [8,30]. A descriptive cross-sectional study among children and adolescents with dog bites in India reported that 96.3% had antibody values ≥0.5 IU/mL on Day 28 after administration of three doses (Days 0, 3, 7) as ID at 0.1 ml in two sites, although antibody testing was performed using an indirect enzyme-linked immunosorbent assay, and not with RFFIT [31]. However, these studies did not provide a randomised head-to-head comparison of IPC and UTRC regimens with extended follow-up using both embryo and Vero-cell-based vaccines. Our trial therefore adds locally generated comparative evidence on the immunogenicity and safety of the IPC regimen in the Indian context.

The National Technical Advisory Group for the Rabies Control Programme (NRCP) has not implemented the IPC regimen in India due to a lack of evidence from local studies. Many countries in Asia and Africa are still using the UTRC regimen. In our study, all participants in both vaccine groups achieved RVNA titres of ≥0.5 IU/mL on Days 14, 28, 42, 90, 180 and 365. These findings provide evidence to support policy consideration of the 1-week abridged IPC regimen in rabies control programs.

From a programmatic perspective, the IPC regimen offers substantial advantages. The UTRC regimen requires 0.8 mL of vaccine, whereas the IPC requires 0.6 mL, which is 25% less for one patient. This, in turn, can enable the treatment of 33% more patients with the same quantity of vaccine [32]. Implementing the abridged regimen in public healthcare settings can save vaccine costs. Additionally, it eliminates one extra visit by the patient for animal bites, thereby increasing compliance with the schedule and reducing travel and other opportunity costs. Currently, anti-rabies vaccines are available in only 80% of public health facilities in India [24]. Introducing the dose-sparing IPC regimen could improve vaccine availability at all health facilities.

Apart from immunogenicity, we also compared the safety of both UTRC and IPC regimens. The safety profile of CCEEVs using IDRV is well established. Safety analysis revealed that adverse events were reported in one-third of the participants across all four arms, which is comparable to other studies on IDRV safety [20]. Only one participant had an SAE, which was unrelated to vaccination.

Our study has certain limitations. First, nearly one-fourth of the participants had an RVNA titre above 0.5 IU/mL at baseline, indicating prior exposure to rabies vaccination. Although the participants with past histories of dog bite or rabies vaccination were excluded during the screening, any anti-rabies vaccination administered during childhood might not have been recalled by the participant. Baseline seropositivity may have influenced GMC estimates because previously primed individuals can mount an anamnestic response after booster vaccination. However, the RVNA titres ≥0.5 IU/mL from Day 14 onward were observed across the study groups irrespective baseline serostatus. Second, the trial was conducted among healthy volunteers, rather than patients with dog bites, which may limit direct generalizability to real-world PEP settings. A randomised comparison of PEP regimens in patients with dog bites could raise ethical concerns because rabies is 100% fatal disease and UTRC is the accepted standard of care in India. Moreover, the use of rabies immunoglobulin, administered along with the vaccine for category III exposures, may attenuate the antibody response and confound immunogenicity outcomes [33]. Third, the trial involved healthy adults, whereas the incidence of bites is more common among children and the elderly [2], whose immune status may differ from that of adults. Fourth, RVNA titres were not measured on Day 7. Therefore, this study cannot assess very early antibody responses before Day 14, a period that may be relevant for rare exposures with short incubation periods.

The WHO-recommended 1-week intradermal IPC regimen did not meet the prespecified equivalence criterion for the day-42 GMR when compared with the 1-month UTRC regimen. However, all participants irrespective of the baseline serostatus achieved RVNA titres ≥0.5 IU/mL from day 14 onward and maintained this response through 1 year, with no major safety concerns. Given its reduced number of visits and lower vaccine requirement, the IPC regimen remains a programmatically attractive shorter alternative to UTRC. Policy decisions regarding replacement of UTRC should consider these immunogenicity findings together with compliance, cost, and vaccine availability.

## Supporting information

S1 Appendix Anti-rabies vaccine schedule of Asian and African countries. (DOCX)

S2 Appendix Geometric mean concentration ratios of rabies virus neutralising antibody titres for pooled IPC and UTRC regimens across PCECV and PVRV recipients: per-protocol and modified intention-to-treat analyses. (DOCX)

S3 Appendix Subject disposition. (DOCX)

S4 Appendix Inclusion/exclusion criteria of the participants. (DOCX)

S5 Appendix Available outcome analysis. (DOCX)

S6 Appendix Rabies virus neutralisation titre thresholds by time points and study arms. (DOCX)

S7 Appendix Protocol. (DOCX)

S1 Checklist CONSORT 2025 checklist of information to include when reporting a randomized trial: Hopewell S, Chan AW, Collins GS, Hróbjartsson A, Moher D, Schulz KF, et al. CONSORT 2025 Statement: updated guideline for reporting randomised trials. BMJ. 2025; 388:e081123. https://dx.doi.org/10.1136/bmj-2024-081123. © 2025 Hopewell et al. This is an Open Access article distributed under the terms of the Creative Commons Attribution License (https://creativecommons.org/licenses/by/4.0/), which permits unrestricted use, distribution, and reproduction in any medium, provided the original work is properly cited.
(DOCX)

## Abbreviations

AEs: adverse events
DMEM: Dulbecco’s’ Modified Eagle Medium
DSMB: Data and Safety Monitoring Board
GMR: geometric mean concentration ratios
IDRV: intradermal rabies vaccination
IPC: Institut Pasteur du Cambodge
MGMCRI: Mahatma Gandhi Medical College and Research Institute
mITT: modified intention-to-treat
NIMHANS: National Institute of Mental Health and Neurosciences
NRCP: National Rabies Control Programme
PCECV: Purified Chick Embryo Cell vaccine
PEP: post-exposure prophylaxis
PVRV: Purified Vero Cell Rabies vaccine
RFFIT: Rapid Fluorescent Focus Inhibition Test
RVIT: Rabies Vaccine Immunogenicity Trial
RVNA: rabies virus neutralising antibodies
SAEs: serious adverse events
TRC: Thai Red Cross
UTRC: Updated Thai Red Cross regimen.
