# Supplementary material for: Immunogenicity and safety of Institut Pasteur du Cambodge (IPC) and updated Thai Red Cross (UTRC) intradermal rabies vaccine regimens among healthy adults in India: A phase 4, open label, equivalence randomised controlled trial
Source: PLoS Med. 2026 Oct 29;23(10):e1005079. doi: 10.1371/journal.pmed.1005079 (PMC13734584; doi:10.1371/journal.pmed.1005079)
Supplement: Supplementary file 1 [file pmed.1005079.s001.docx]

**S1 Appendix:** Anti-rabies vaccine schedule of Asian and African countries

| **S. No** | **Country** | **Prophylaxis** | **Doses** | **Route of Administration** | **Vaccine Schedule** | **Source** |
| --- | --- | --- | --- | --- | --- | --- |
| 1 | Srilanka | PEP | 0.2 mL per visit | ID | D0,3,7,30 | <http://www.rabies.gov.lk/com/circulars/Anti%20Rabies%20post%20exposure%20therapy%20(PET)%20-%202019.pdf> |
|  |  |  | 0.1 mL * 4 sites on D0 then 0.2 mL per visit | ID | D0,3,7,30 |  |
|  |  |  | 1 full vial or 0.5 mL per visit | IM | D0,3,7,14,30 |  |
|  |  |  | 1 full vial or 0.5 mL * 2 sites on D0 then single dose per visit | IM | D0,7,21 |  |
| 2 | Pakistan | PEP | 1 full vial per visit | IM | D0,3,7,14,28 | <https://mmidsp.com/wp-content/uploads/2019/09/Guidelines-for-rabies-prevention.pdf> |
|  |  |  | 2 full vails on D0 then 1 vial per visit | IM | D0,7,21 |  |
|  |  |  | 0.2 mL per visit | ID | D0,3,7,28 |  |
| 3 | Philippines | PEP | 0.2 mL per visit | ID | D0,3,7,28 | <https://psmid.org/wp-content/uploads/2025/09/CPG-rabies-AO-2018-0013.pdf> |
|  |  |  | 0.2 mL per visit | ID | D0,3,7 |  |
|  |  |  | 0.5 mL or 1 full vial * 2 sites on D0 then single dose per visit | IM | D0,7,21 |  |
|  |  |  | 1 full vial or 0.5 mL per visit | IM | D0,3,7,14 |  |
| 4 | Indonesia | PEP | 0.5 mL * 2 sites on D0 then single dose per visit | IM | D0,7,21 | <https://www.nitag-resource.org/sites/default/files/8fd7afd3a72bdacb5ac26d27c3d9aae5b96c44b9_1.pdf> |
|  |  |  | 0.2 mL per visit | ID | D0,3,7,28 |  |
| 5 | Taiwan | PEP | 1 mL per visit | IM | D0,3,7,14 | Taiwan follows cdc guidelines for ARV |
| 6 | China | PEP | 1 dose per visit | IM | D0,3,7,14,28 | [https://pmc.ncbi.nlm.nih.gov/articles/PMC12136364/ https://www.sciencedirect.com/science/article/pii/S0264410X22007848](https://pmc.ncbi.nlm.nih.gov/articles/PMC12136364/) |
|  |  |  | 2 Dose on D0, then single dose per visit | IM | D0,7,21 |  |
| 7 | South Korea | PEP | 1 full dose per visit | IM | D0,3,7,14,28 |  |
| 8 | Vietnam | PEP | 0.2 mL per visit | ID | D0,3,7,28 | <https://rr-asia.woah.org/app/uploads/2020/03/06-_post-exposure_prophylaxis_access_final.pdf> |
|  |  |  | 1 full vial per visit | IM | D0,3,7,14,28 |  |
| 9 | Libya | PEP | 1 dose per visit | IM | D0,3,7,14,28 |  |
|  |  |  | 2 Dose on D0, then single dose per visit | IM | D0,7,21 |  |
| 10 | Ethiopia | PEP | Variable by age (e.g., 2–5 mL daily) | Subcutaneous (SC), around umbilicus or other sites | Daily injections for 14 consecutive days + boosters on days 10, 20, 30 after last dose (age-based dosing: up to 2 mL for infants, increasing with age) | <https://ephi.gov.et/wp-content/uploads/2022/08/NTV-Guideline-final-2022.pdf> |
|  |  |  | 0.5 mL or 1 mL per visit | IM | D0,3,7,14,28 & D0,7,21 |  |
| 11 | Guinea | PEP | 0.5 mL or 1 mL per visit | IM | D0,3,7,14,28 | Touré A, Savadogo M, Doumbouya MI, Kourouma F, Gbamou P, Tarnagda Z, Bada-Alambedji R. Rabies surveillance and prevention in Guinea: Epidemiological data and postexposure prophylaxis challenges. Vet World. 2024 Aug;17(8):1828-1835. doi: 10.14202/vetworld.2024.1828-1835. Epub 2024 Aug 20. PMID: 39328452; PMCID: PMC11422633. |
|  |  |  | 2 Dose on D0, then single dose per visit | IM | D0,7,21 |  |
| 12 | Kenya | PEP | 0.5 mL or 1 mL per visit | IM | D0,3,7,14,28 | <http://guidelines.health.go.ke:8000/media/Kenya_National_Immunization_Policy_Guidelines_Version_signed.pdf> |
|  |  |  | 0.2 mL per visit | ID | D0,3,7,28 |  |
| 13 | Morocco | PEP | 2 Dose on D0, then single dose per visit | IM | D0,7,21 | As per Morocco national program, zagreb is the preferred regimen/ https://www.slideshare.net/slideshow/programme-national-de-lutte-contre-la-rage/59828496 |
| 14 | Madagascar | PEP | 0.2 mL per visit | ID | D0,3,7,28 | Rajeev M, Guis H, Edosoa GT, Hanitriniaina C, Randrianarijaona A, Mangahasimbola RT, Hierink F, Ramiandrasoa R, Nely J, Heraud JM, Andriamandimby SF, Baril L, Metcalf CJE, Hampson K. How geographic access to care shapes disease burden: The current impact of post-exposure prophylaxis and potential for expanded access to prevent human rabies deaths in Madagascar. PLoS Negl Trop Dis. 2021 Apr 26;15(4):e0008821. doi: 10.1371/journal.pntd.0008821. PMID: 33901194; PMCID: PMC8102000. |
| 15 | Uganda | PEP | 2 Dose on D0, then single dose per visit | IM | D0,7,21 | <https://hope.co.ug/rabies-vaccine-schedules-in-uganda/> |
|  |  |  | 0.2 mL per visit | ID | D0,3,7,28 |  |
| 16 | Sudan | PEP | 0.5 mL or 1 mL per visit | IM | D0,3,7,14,28 | Shaban MAA, Sidahmed TSM, Elobied HES, Omer MME, Yusuf AAA, El-Haj AMOK, Yassin MFA, Abbas HEM, AbdAlrhman FAM, Ahmed OEM. Assessment of the awareness of rabies, rabies prophylaxis guidelines and rabies practice among physicians in Sudan: a national cross-sectional study, 2024. BMC Public Health. 2025 Mar 6;25(1):892. doi: 10.1186/s12889-025-21949-4. PMID: 40050827; PMCID: PMC11884061. |
|  |  |  | 2 Dose on D0, then single dose per visit | IM | D0,7,21 |  |
|  |  |  | 0.2 mL per visit | ID | D0,3,7,28 |  |
