# Supplementary material for: Immunogenicity and safety of Institut Pasteur du Cambodge (IPC) and updated Thai Red Cross (UTRC) intradermal rabies vaccine regimens among healthy adults in India: A phase 4, open label, equivalence randomised controlled trial
Source: PLoS Med. 2026 Oct 29;23(10):e1005079. doi: 10.1371/journal.pmed.1005079 (PMC13734584; doi:10.1371/journal.pmed.1005079)
Supplement: Supplementary file 2 [file pmed.1005079.s002.docx]

**S2 Appendix**: Geometric mean concentration ratios of rabies virus neutralising antibody titres for pooled IPC and UTRC regimens across PCECV and PVRV recipients: per-protocol and modified intention-to-treat analyses

| **Blood Sample collection at Scheduled Visits** | **Abridged** | **Updated** |  | **Abridged** | **Updated** |  |
| --- | --- | --- | --- | --- | --- | --- |
|  | **Per Protocol Analysis** | | | **ITT Analysis** | | |
|  | **n = 184** | **n =178** |  | **n = 193** | **n = 182** |  |
|  | Geometric Mean (SD) | Geometric Mean (SD) | Geometric Mean Ratio (GMR) 95% CI | Geometric Mean (SD) | Geometric Mean (SD) | Geometric Mean Ratio (GMR) 95% CI |
| Day 0 | 0.36 (7.23) | 0.35 (7.00) | 1.01(0.67 - 1.52) | 0.36 (7.21) | 0.36 (7.08) | 1.00(0.67 - 1.49) |
| Day 14 | 16.06 (2.60) | 16.90 (2.39) | 0.95(0.79 - 1.15) | 16.06 (2.59) | 16.82 (2.38) | 0.95(0.79 - 1.15) |
| Day 28 | 16.12 (2.38) | 15.15 (2.31) | 1.06(0.89 - 1.27) | 15.89 (2.38) | 15.05 (2.33) | 1.06(0.89 - 1.26) |
| Day 42 | 13.71 (2.33) | 19.90 (2.09) | 0.69(0.58 - 0.81) | 13.32 (2.34) | 19.80 (2.10) | 0.67(0.57 - 0.79) |
| Day 90 | 7.36 (2.94) | 16.58 (2.51) | 0.44(0.36 - 0.55) | 7.18 (2.95) | 16.50 (2.50) | 0.44(0.35 - 0.53) |
| Day 180 | 5.29 (2.74) | 6.79 (2.55) | 0.78(0.64 - 0.95) | 5.24 (2.72) | 6.84 (2.54) | 0.77(0.63 - 0.93) |
| Day 365 | 6.04 (2.69) | 7.23 (2.48) | 0.84(0.69 - 1.02) | 5.98 (2.65) | 7.24 (2.48) | 0.83(0.68 - 1.00) |
|  |  |  |  |  |  |  |
| Sero-Negative Subjects | n = 142 | n = 137 |  | n = 149 | n = 140 |  |
| Day 0 | - | - | - | - | - | - |
| Day 14 | 11.94 (2.15) | 12.94 (2.08) | 0.92(0.77 - 1.10) | 12.03 (2.14) | 12.93 (2.06) | 0.93(0.78 - 1.11) |
| Day 28 | 12.00 (2.02) | 11.29 (1.93) | 1.06(0.91 - 1.25) | 11.97 (2.01) | 11.15 (1.94) | 1.07(0.92 - 1.26) |
| Day 42 | 11.04 (2.10) | 16.91 (1.99) | 0.65(0.55 - 0.77) | 10.77 (2.10) | 16.73 (1.99) | 0.64(0.55 - 0.76) |
| Day 90 | 5.41 (2.69) | 13.44 (2.44) | 0.40(0.32 - 0.50) | 5.25 (2.69) | 13.35 (2.42) | 0.39(0.32 - 0.49) |
| Day 180 | 3.61 (2.03) | 5.07 (2.16) | 0.71(0.60 - 0.85) | 3.58 (2.02) | 5.12 (2.15) | 0.70(0.59 - 0.83) |
| Day 365 | 4.37 (2.13) | 5.54 (2.19) | 0.79(0.66 - 0.94) | 4.37 (2.11) | 5.56 (2.19) | 0.79(0.66 - 0.94) |
|  |  |  |  |  |  |  |
| Sero-Positive Subjects | n = 42 | n = 41 |  | n = 44 | n = 42 |  |
| Day 0 | 12.49 (2.31) | 11.41 (2.27) | 1.09(0.76 - 1.57) | 12.43 (2.33) | 11.69 (2.29) | 1.06(0.74 - 1.52) |
| Day 14 | 43.79 (2.32) | 41.24 (2.03) | 1.06(0.76 - 1.49) | 41.83 (2.46) | 41.24 (2.03) | 1.01(0.71 - 1.44) |
| Day 28 | 43.79 (1.78) | 40.54 (1.81) | 1.08(0.84 - 1.39) | 41.17 (1.95) | 40.99 (1.80) | 1.00(0.77 - 1.32) |
| Day 42 | 28.51 (2.15) | 34.24 (1.89) | 0.83(0.61 - 1.13) | 27.34 (2.23) | 34.75 (1.89) | 0.79(0.58 - 1.07) |
| Day 90 | 20.83 (1.88) | 33.10 (1.89) | 0.63(0.48 - 0.83) | 20.59 (1.86) | 33.07 (1.88) | 0.62(0.48 - 0.81) |
| Day 180 | 19.18 (2.15) | 18.01 (2.20) | 1.07(0.76 - 1.50) | 19.03 (2.12) | 17.96 (2.18) | 1.06(0.76 - 1.47) |
| Day 365 | 19.03 (2.35) | 17.41 (2.08) | 1.09(0.77 - 1.55) | 18.26 (2.39) | 17.38 (2.06) | 1.05(0.74 - 1.49) |
