# Supplementary material for: Immunogenicity and safety of Institut Pasteur du Cambodge (IPC) and updated Thai Red Cross (UTRC) intradermal rabies vaccine regimens among healthy adults in India: A phase 4, open label, equivalence randomised controlled trial
Source: PLoS Med. 2026 Oct 29;23(10):e1005079. doi: 10.1371/journal.pmed.1005079 (PMC13734584; doi:10.1371/journal.pmed.1005079)
Supplement: Supplementary file 3 [file pmed.1005079.s003.docx]

**S3 Appendix: Subject disposition**

| Trial Subjects Disposition | n | % |
| --- | --- | --- |
| No. of Subjects screened for trial eligibility | 418 |  |
| No. of Subjects did not meet eligibility (Inclusion / Exclusion Criteria) | 18 | 4.3 |
| No. of the Subjects did not meet the inclusion criteria | 7 |  |
| The study participant's health status (As decided by the Study Physician) | 7 |  |
| No. of Subjects Met Exclusion Criteria | 6 |  |
| History of Dog Bite / Vaccination | 5 |  |
| Treatment with parenteral or oral steroids /immunosuppressive drugs during the last two months | 1 |  |
| Temporarily Ineligible due to any acute conditions | 5 |  |
| No. of Eligible Subjects Randomized | 400 | 95.7 |
| No. of Eligible Subjects Participation Status |  |  |
| Completed the study | 377 | 94.3 |
| Did not complete the study | 23 | 5.8 |
| Lost to follow-up | 11 |  |
| Out-migration | 4 |  |
| Others (Unwilling for participation/blood draw) | 8 |  |
| No. of Randomized Subjects considered for Outcome Analysis |  |  |
| Per Protocol Population (Window Period Visits - 5, Vaccine Dose - 3 or 4, Blood/Lab results - 4) | 362 | 96.0 |
| Intention to Treat Population | 375 | 99.5 |
| Missed Visits/ Out of Window Period Visits | 13 |  |
| Missed Blood/ Lab Results for Day-42 | 2 |  |
