# Supplementary material for: Immunogenicity and safety of Institut Pasteur du Cambodge (IPC) and updated Thai Red Cross (UTRC) intradermal rabies vaccine regimens among healthy adults in India: A phase 4, open label, equivalence randomised controlled trial
Source: PLoS Med. 2026 Oct 29;23(10):e1005079. doi: 10.1371/journal.pmed.1005079 (PMC13734584; doi:10.1371/journal.pmed.1005079)
Supplement: Supplementary file 4 [file pmed.1005079.s004.docx]

**S4 Appendix: Inclusion/exclusion criteria of the participants**

Inclusion Criteria

1. Is the study participant healthy

2. Study participants are aged between 18-45 years

3. Study participant willing to provide informed consent

4. Study participants are willing to give blood sample on (Days 0,14,28,42,180 and 365)

Exclusion Criteria

1. Presence of fever (> 99.3°F) on the day of enrolment (temporary exclusion).

2. Acute disease at the time of enrolment (temporary exclusion)

3. Concurrent participation in another clinical trial throughout the entire timeframe for this study.

4. History of dog bite

5. History of rabies immunization

6. Anti-malarial drug treatment during the last two months

7. Treatment with parenteral or oral steroids /immunosuppressive drugs during the last two

months

8. Receiving immunoglobulin

9. Suffering from an autoimmune disease or any other immunological disorder

10. History of hypersensitivity to any vaccine

11. History of drug abuse or alcohol abuse

12. Psychological condition rendering the volunteer unable to understand the nature, scope and

consequence of the study

13. Participation in other investigational trials at the same time

14. Pregnant women and lactating mothers
