# Supplementary material for: Immunogenicity and safety of Institut Pasteur du Cambodge (IPC) and updated Thai Red Cross (UTRC) intradermal rabies vaccine regimens among healthy adults in India: A phase 4, open label, equivalence randomised controlled trial
Source: PLoS Med. 2026 Oct 29;23(10):e1005079. doi: 10.1371/journal.pmed.1005079 (PMC13734584; doi:10.1371/journal.pmed.1005079)
Supplement: Supplementary file 5 [file pmed.1005079.s005.docx]

# **S5 Appendix: Available outcome analysis**

| Blood Collection at Scheduled Visits | PCECV IPC Regimen (Day 0,3,7) | | PCECV UTRC Regimen (Day 0,3,7,28) | | Geometric Mean Ratio (GMR) (95% CI) | PVRV IPC Regimen (Day 0,3,7) | | PVRV UTRC  Regimen (Day 0,3,7,28) | | Geometric Mean Ratio (GMR) (95% CI) |
| --- | --- | --- | --- | --- | --- | --- | --- | --- | --- | --- |
|  | **n** | **Geometric Mean (GSD)**  **IU/ml** | **n** | **Geometric Mean (GSD)**  **IU/ml** |  | **n** | **Geometric Mean (GSD)**  **IU/ml** | **n** | **Geometric Mean (GSD)**  **IU/ml** |  |
| Day 0 | 100 | 0.34 (6.69) | 100 | 0.29 (5.82) | 1.16(0.70 - 1.94) | 100 | 0.36 (7.61) | 100 | 0.39 (7.83) | 0.93(0.52 - 1.64) |
| Day 14 | 95 | 15.43 (2.70) | 91 | 17.13 (2.33) | 0.90(0.69 - 1.18) | 96 | 16.71 (2.48) | 93 | 16.24 (2.45) | 1.03(0.79 - 1.33) |
| Day 28 | 95 | 14.66 (2.56) | 90 | 14.04 (2.32) | 1.04(0.81 - 1.35) | 98 | 17.05 (2.19) | 93 | 16.00 (2.34) | 1.07(0.84 - 1.35) |
| Day 42 | 96 | 12.25 (2.45) | 89 | 20.69 (2.10) | 0.59(0.47 - 0.75) | 97 | 14.48 (2.23) | 93 | 18.99 (2.10) | 0.76(0.61 - 0.95) |
| Day 90 | 96 | 6.92 (2.97) | 91 | 17.00 (2.49) | 0.41(0.30 - 0.54) | 97 | 7.34 (2.95) | 92 | 15.41 (2.54) | 0.48(0.36 - 0.64) |
| Day 180 | 96 | 5.30 (2.65) | 91 | 6.77 (2.55) | 0.78(0.59 - 1.03) | 98 | 5.16 (2.79) | 94 | 6.85 (2.54) | 0.75(0.57 - 1.00) |
| Day 365 | 96 | 6.21 (2.69) | 90 | 6.91 (2.69) | 0.90(0.68 - 1.20) | 96 | 5.82 (2.62) | 94 | 7.43 (2.30) | 0.78(0.61 - 1.01) |
| Sero-Negative Subjects | | | | | | | | | | |
| Day 0 | 77 | - | 80 | - | - | 78 | - | 76 | - | - |
| Day 14 | 73 | 12.15 (2.31) | 72 | 13.20 (1.97) | 0.92(0.72 - 1.18) | 74 | 11.97 (1.97) | 71 | 12.53 (2.18) | 0.95(0.75 - 1.21) |
| Day 28 | 73 | 11.05 (2.21) | 71 | 10.51 (1.91) | 1.05(0.83 - 1.33) | 76 | 12.85 (1.80) | 70 | 11.77 (1.96) | 1.09(0.89 - 1.34) |
| Day 42 | 74 | 9.83 (2.23) | 70 | 18.02 (2.03) | 0.55(0.43 - 0.70) | 75 | 11.79 (1.97) | 70 | 15.53 (1.94) | 0.76(0.61 - 0.95) |
| Day 90 | 74 | 5.06 (2.72) | 72 | 13.85 (2.41) | 0.37(0.27 - 0.50) | 75 | 5.38 (2.67) | 69 | 12.32 (2.46) | 0.44(0.32 - 0.60) |
| Day 180 | 74 | 3.71 (1.98) | 72 | 5.04 (2.01) | 0.74(0.59 - 0.92) | 76 | 3.46 (2.05) | 71 | 5.21 (2.32) | 0.66(0.51 - 0.86) |
| Day 365 | 74 | 4.48 (2.10) | 71 | 5.31 (2.38) | 0.84(0.65 - 1.10) | 76 | 4.34 (2.15) | 71 | 5.74 (2.04) | 0.76(0.59 - 0.96) |
| Blood Collection at Scheduled Visits | **PCECV IPC Regimen (Day 0,3,7)** | | **PCECV UTRC Regimen (Day 0,3,7,28)** | | **Geometric Mean Ratio (GMR) (95% CI)** | **PVRV IPC Regimen (Day 0,3,7)** | | **PVRV UTRC  Regimen (Day 0,3,7,28)** | | **Geometric Mean Ratio (GMR) (95% CI)** |
|  | **n** | **Geometric Mean (GSD)**  **IU/ml** | **n** | **Geometric Mean (GSD)**  **IU/ml** |  | **n** | **Geometric Mean (GSD)**  **IU/ml** | **n** | **Geometric Mean (GSD)**  **IU/ml** |  |
| Sero-Positive Subjects | | | | | | | | | | |
| Day 0 | 23 | 9.88 (2.57) | 20 | 8.88 (2.55) | 1.11(0.62 - 1.99) | 22 | 15.50 (1.93) | 24 | 14.25 (1.95) | 1.09(0.73 - 1.61) |
| Day 14 | 22 | 34.08 (2.91) | 19 | 46.09 (1.95) | 0.74(0.42 - 1.31) | 22 | 51.33 (1.93) | 22 | 37.46 (2.09) | 1.37(0.90 - 2.10) |
| Day 28 | 22 | 37.46 (2.16) | 19 | 41.31 (1.78) | 0.91(0.59 - 1.40) | 22 | 45.25 (1.74) | 23 | 40.72 (1.85) | 1.11(0.78 - 1.58) |
| Day 42 | 22 | 25.67 (2.25) | 19 | 34.42 (1.91) | 0.75(0.47 - 1.19) | 22 | 29.11 (2.25) | 23 | 35.03 (1.89) | 0.83(0.54 - 1.29) |
| Day 90 | 22 | 19.95 (1.79) | 19 | 37.03 (1.73) | 0.54(0.38 - 0.77) | 22 | 21.24 (1.94) | 23 | 30.13 (1.99) | 0.71(0.47 - 1.06) |
| Day 180 | 22 | 17.59 (2.31) | 19 | 20.66 (2.46) | 0.85(0.49 - 1.47) | 22 | 20.59 (1.94) | 23 | 16.00 (1.94) | 1.29(0.86 - 1.91) |
| Day 365 | 22 | 18.73 (2.54) | 19 | 18.51 (2.20) | 1.01(0.58 - 1.75) | 20 | 17.75 (2.27) | 23 | 16.49 (1.97) | 1.08(0.67 - 1.72) |
