# Supplementary material for: Immunogenicity and safety of Institut Pasteur du Cambodge (IPC) and updated Thai Red Cross (UTRC) intradermal rabies vaccine regimens among healthy adults in India: A phase 4, open label, equivalence randomised controlled trial
Source: PLoS Med. 2026 Oct 29;23(10):e1005079. doi: 10.1371/journal.pmed.1005079 (PMC13734584; doi:10.1371/journal.pmed.1005079)
Supplement: Supplementary file 6 [file pmed.1005079.s006.docx]

**S6 Appendix: Rabies virus neutralization titer thresholds by time points and study arms**

| Blood Collection at Scheduled Visits | RVNA Titer Threshold | PCECV IPC Regimen (Day 0,3,7) | PCECV UTRC Regimen (Day 0,3,7,28) | PVRV IPC Regimen (Day 0,3,7) | PVRV UTRC  Regimen (Day 0,3,7,28) | Total |
| --- | --- | --- | --- | --- | --- | --- |
| Day 0 |  | n = 100 | n = 100 | n = 100 | n = 100 | n = 400 |
|  | < 0.5 | 77 | 80 | 78 | 76 | 311 |
|  | 2 | 4 | 3 | 0 | 1 | 8 |
|  | 4 | 1 | 3 | 1 | 1 | 6 |
|  | 8 | 6 | 6 | 7 | 4 | 23 |
|  | 16 | 9 | 4 | 6 | 13 | 32 |
|  | 32 | 2 | 4 | 8 | 5 | 19 |
|  | 64 | 1 | 0 | 0 | 0 | 1 |
| Day 14 |  | n = 95 | n = 91 | n = 96 | n = 93 | n = 375 |
|  | 0.5 | 1 | 0 | 0 | 0 | 1 |
|  | 2 | 2 | 0 | 0 | 2 | 4 |
|  | 4 | 11 | 6 | 8 | 9 | 34 |
|  | 8 | 20 | 24 | 29 | 22 | 95 |
|  | 16 | 31 | 34 | 29 | 27 | 121 |
|  | 32 | 13 | 12 | 13 | 18 | 56 |
|  | 64 | 16 | 12 | 14 | 15 | 57 |
|  | 128 | 1 | 3 | 2 | 0 | 6 |
|  | >128 | 0 | 0 | 1 | 0 | 1 |
| Day 28 |  | n = 95 | n = 90 | n = 98 | n = 93 | n = 376 |
|  | 4 | 17 | 11 | 5 | 8 | 41 |
|  | 8 | 26 | 30 | 26 | 28 | 110 |
|  | 16 | 19 | 26 | 37 | 28 | 110 |
|  | 32 | 19 | 12 | 17 | 16 | 64 |
|  | 64 | 13 | 10 | 11 | 11 | 45 |
|  | 128 | 1 | 1 | 2 | 2 | 6 |
| Day 42 |  | n = 96 | n = 89 | n = 97 | n = 93 | n = 375 |
|  | 2 | 5 | 0 | 1 | 0 | 6 |
|  | 4 | 13 | 4 | 9 | 6 | 32 |
|  | 8 | 26 | 13 | 29 | 14 | 82 |
|  | 16 | 31 | 33 | 31 | 36 | 131 |
|  | 32 | 13 | 24 | 19 | 25 | 81 |
|  | 64 | 7 | 15 | 7 | 12 | 41 |
|  | 128 | 1 | 0 | 1 | 0 | 2 |
|  |  | n = 90 | n = 91 | n = 95 | n = 92 | n = 368 |
| Day 90 | 0.5 | 0 | 0 | 1 | 0 | 1 |
|  | 2 | 17 | 3 | 23 | 4 | 47 |
|  | 4 | 20 | 6 | 13 | 7 | 46 |
|  | 8 | 18 | 24 | 18 | 25 | 85 |
|  | 16 | 22 | 18 | 26 | 25 | 91 |
|  | 32 | 10 | 28 | 11 | 16 | 65 |
|  | 64 | 2 | 11 | 3 | 15 | 31 |
|  | 128 | 1 | 1 | 0 | 0 | 2 |
| Blood Collection at Scheduled Visits | **RVNA Titer Threshold** | **PCECV IPC Regimen (Day 0,3,7)** | **PCECV UTRC Regimen (Day 0,3,7,28)** | **PVRV IPC Regimen (Day 0,3,7)** | **PVRV UTRC  Regimen (Day 0,3,7,28)** | **Total** |
|  |  | n = 96 | n = 91 | n = 98 | n = 93 | n = 378 |
| Day 180 | 0.5 | 0 | 0 | 1 | 0 | 1 |
|  | 2 | 33 | 16 | 36 | 16 | 101 |
|  | 4 | 26 | 31 | 23 | 30 | 110 |
|  | 8 | 14 | 16 | 13 | 15 | 58 |
|  | 16 | 13 | 19 | 17 | 21 | 70 |
|  | 32 | 8 | 5 | 4 | 10 | 27 |
|  | 64 | 2 | 4 | 4 | 1 | 11 |
| Day 365 |  | n = 95 | n = 88 | n = 95 | n = 94 | n = 372 |
|  | 0.5 | 1 | 1 | 2 | 0 | 4 |
|  | 2 | 20 | 13 | 19 | 12 | 64 |
|  | 4 | 26 | 28 | 26 | 29 | 109 |
|  | 8 | 23 | 11 | 29 | 17 | 80 |
|  | 16 | 16 | 28 | 10 | 30 | 84 |
|  | 32 | 7 | 4 | 6 | 5 | 22 |
|  | 64 | 0 | 3 | 3 | 1 | 7 |
|  | 128 | 2 | 0 |  | 0 | 2 |
