# Supplementary material for: Immunogenicity and safety of Institut Pasteur du Cambodge (IPC) and updated Thai Red Cross (UTRC) intradermal rabies vaccine regimens among healthy adults in India: A phase 4, open label, equivalence randomised controlled trial
Source: PLoS Med. 2026 Oct 29;23(10):e1005079. doi: 10.1371/journal.pmed.1005079 (PMC13734584; doi:10.1371/journal.pmed.1005079)
Supplement: Supplementary file 7 [file pmed.1005079.s007.docx]

# **S7 Appendix: Protocol (Version & Date:3.1 & 29.12.2023)**

**Immunogenicity of rabies vaccine using an abridged Cambodia regimen and updated Thai Red Cross regimen in healthy volunteers in India: A parallel multicentric randomised open label trial.**

| **S.no** | **Version & Date** | **Approval** | **Amendment details** |
| --- | --- | --- | --- |
| 1 | 2.0 & 04.10.2021 | Approved by IHEC on 27.10.2021 | First approval |
| 2 | 2.1 & 31.05.2023 | Approved by IHEC on 05.06.2023 | Study participants have been changed from healthy volunteers of veterinary college students & conservancy workers to healthy volunteers of medical/para medical/allied health science college students.  The age of study participants has been changed from 18-30 years to 18-45 years |
| 3 | 3.0 & 13.09.2023 | Approved by IHEC on 20.09.2023 | Sample collection on day 90 was added.  Changes to the sample size assumptions were made. |
| 4 | 3.1 & 29.12.2023 | Approved by IHEC on 24.01.2024 | Changed the testing site from NIMHANS, Bengaluru, to ICMR-NIV, Pune. |

1. **Summary:**

Rabies is a fatal, viral zoonotic disease causing an estimated 59,000 annual human deaths globally. Effective treatment soon after exposure to rabies can prevent the onset of symptoms and death. A recent multicentric community survey conducted in seven states of India on animal bites in human, showed post exposure prophylaxis coverage of 79.6%. The National Rabies Control Programme of India (NRCP) recommends Updated Thai Red Cross regimen (2-2-2-0-2) for animal bite cases and this regimen is implemented in India. WHO Expert consultation on Rabies in 2018 recommended a short one-week intradermal vaccination course known as Institute Pasteur, Cambodia regimen (2-2-2-0-0). Abridged anti-rabies vaccination schedule can reduce costs and improve compliance to the schedule. To implement the new WHO recommendation of abridged Cambodia regimen (2-2-2-0-0), sufficient evidence is necessary pertaining to Indian settings. In this regard we propose to compare the safety and immunogenicity of the abridged Cambodia regimen (2-2-2-0-0) with updated Thai red cross regimen (2-2-2-0-2) in healthy volunteers through a multicentric randomised open label trial.

2. **Keywords:** Rabies vaccination, Immunogenicity, Intradermal Vaccination, Cambodia regimen, Thai red cross regimen,
3. **Background and rationale:**

Rabies is one of the oldest and high fatal viral zoonotic diseases causing an estimated 59,000 annual human deaths globally, with most deaths occurring in Asia and Africa (1). WHO, the World Organisation for Animal Health (OIE), the Food and Agriculture Organization of the United Nations (FAO) and the Global Alliance for Rabies Control (GARC) have established a global “United Against Rabies” collaboration to provide a common strategy to achieve "Zero human rabies deaths by 2030". An estimated 20,000 rabies deaths (approximately 2/100,000 population) and 17.4 million exposures to animal bite occur every year in India (2,3).

Effective treatment soon after exposure to rabies can prevent the onset of symptoms and death. Enhancing availability and accessibility of anti-rabies vaccines can reduce deaths due to rabies. A recent multicentric community survey conducted in seven states of India on animal bites in human, showed post exposure prophylaxis coverage of 79.6% (4). Efforts are required to improve access to adequate and timely rabies post-exposure prophylaxis. But the compliance was an issue with earlier WHO-recommended regimens requiring five visits over 1-month period. Reducing the number of visits by providing a shortened regimen will reduce direct and indirect costs and improve access. (5). In this context WHO Expert consultation on Rabies in 2018 recommended a short one-week intradermal vaccination course known as Institute Pasteur, Cambodia regimen (2-2-2-0-0) (5).

Newer vaccines in use today cause fewer adverse reactions than previously available vaccines. Mild, local reactions to the rabies vaccine, such as pain, redness, swelling, or itching at the injection site, have been reported. Rarely, symptoms such as headache, nausea, abdominal pain, muscle aches, and dizziness have been reported. Local pain and low-grade fever may follow injection of rabies immune globulin. The vaccine should be given at recommended intervals for best results (6).

Pre-exposure, active immunization can be achieved by administration of safe and potent culture vaccines. Pre-exposure vaccination is highly recommended by the NRCP to at risk groups like laboratory staff handling the rabies virus and infective material, clinicians and persons attending to human rabies cases, veterinarians, animal handlers and catchers, wild life wardens, quarantine officers and travellers from rabies free areas to rabies endemic areas (7). The first dose is given on any chosen day (counted as day zero), The second dose should be given seven days later, the third dose should be given 21 or 28 days after the first dose. The pre-exposure prophylaxis is given through intradermal route as 0.1 ml at one site. For those individuals who continue to be at increased risk of contracting rabies based on the advice of the rabies expert or infectious diseases specialist, booster doses of vaccine are recommended every two years or at suitable intervals to maintain protective antibody levels in such individuals.

Currently National Rabies Control Programme of India (NRCP) recommend Updated Thai Red Cross regimen (2-2-2-0-2). It’s a 2-site intradermal method with a dose of 0.1ml per site. One dose per site must be given intradermally on Days 0, 3, 7, 28. In order to implement the new WHO recommendation of abridged Cambodia regimen (2-2-2-0-0), (2-site intradermal method with a dose of 0.1ml per site must be given on Days 0, 3, 7). Sufficient evidence is necessary pertaining to Indian settings. In this regard we propose to compare the safety and immunogenicity of the abridged Cambodia regimen (2-2-2-0-0) with updated Thai red cross regimen (2-2-2-0-2) in healthy volunteers through a randomised open label trial.

4. **Novelty/Innovation:**

A Parallel group randomised open label trial will be conducted to determine the safety and immunogenicity of the Intradermal abridged Cambodia regimen. The evidence from the study will guide the decision makers to decide on implementing WHO 2018 recommendation on abridged Cambodia regimen on India. The shortened course would improve patient compliance to the schedule, reduce direct and indirect costs to the patient as well as for the NRCP of India.

5. **Study Hypothesis**

Anti-Rabies vaccination using intradermal abridged Cambodia regimen (2-2-2-0-0) is equivalent in immunogenicity to intradermal updated Thai red cross regimen (2-2-2-0-2) among two different vaccine types. Thus, the study will test two hypotheses.

| Hypothesis 1 | Purified Verocell Rabies Vaccine | Abridged Cambodia regimen |
| --- | --- | --- |
|  |  | Updated Thai red cross regimen |
| Hypothesis 2 | Purified Chick Embryo Cell Vaccine | Abridged Cambodia regimen |
|  |  | Updated Thai red cross regimen |

6. **Study objectives**:

- Determine immunogenicity of the Intradermal abridged Cambodia regimen (2-2-2-0-0) with updated intradermal Thai red cross regimen (2-2-2-0-2) using two different vaccine types among healthy volunteers in India.
- Describe safety profile of the Intradermal abridged Cambodia regimen (2-2-2-0-0) with updated intradermal Thai red cross regimen (2-2-2-0-2) using two different vaccine types

7. **Study Design:**

The study is designed as an open label, randomized, equivalence clinical trial with four parallel groups of healthy volunteers receiving either abridged Cambodia regimen or updated Thai cross regimen using purified verocell Rabies vaccine and purified chick embryo vaccine as anti-rabies vaccination

8. **Study participants**

The study population will be among Healthy Volunteers and Medical /Para medical/ Allied health science college students of Sri Balaji Vidyapeeth (SBV) University in Puducherry and Chennai

9**. Eligibility criteria**

9.1. **Inclusion criteria**

A signed informed written witnessed consent will be obtained from each volunteer before initiating any study-related activity.

a) Healthy males or females
b) Aged between 18 to 45 years
c) Willing to give blood sample on Days 0, 14, 28, ,90, 42, 180 and 365.

9.2. **Exclusion criteria**

a) History of dog bite
b) History of rabies immunization
c) Anti-malarial drug treatment during the last two months
d) Treatment with parenteral or oral steroids / immunosuppressive drugs during the last two months
e) Receiving immunoglobulins
f) Suffering from an autoimmune disease or any other immunological disorder
g) History of hypersensitivity to any vaccine
h) History of drug abuse or alcohol abuse
i) Psychological condition rendering the volunteer unable to understand the nature, scope and consequence of the study
j) Participation in other investigational trials at the same time
k) Pregnant women and lactating mothers

9.3. **Study groups**

We have planned to enrol study participants four study arms. They are

1. Purified Verocell Rabies Vaccine – Abridged Cambodia regimen
2. Purified Verocell Rabies Vaccine – Updated Thai red cross regimen
3. Purified Chick Embryo Cell Vaccine – Abridged Cambodia regimen
4. Purified Chick Embryo Cell Vaccine – Updated Thai red cross regimen
10. **Interventions:**

Two vaccine types

- Purified Verocell Rabies Vaccine
- Purified Chick Embryo Cell Vaccine

**Abridged Cambodia Vaccination schedule**

- WHO-recommended 2-site intradermal method with a dose of 0.1ml per site will be adopted. One dose per site must be given intradermally on Days 0, 3, 7. Its given in the deltoid region of the arm

**Updated Thai red cross Vaccination schedule**

- It’s a 2-site intradermal method with a dose of 0.1ml per site will be adopted. One dose per site must be given intradermally on Days 0, 3, 7, 28. Its given in the deltoid region of the arm

We will use vaccines that are WHO prequalified vaccine and routinely used in

the programme such as Rabivax-S of SIIL and ChiroRab® by Chiron Behring Vaccines Private Ltd.

11. **Outcome**

Primary outcome:

- The Primary outcome measure will be geometric mean titres of Rabies Virus Neutralizing Antibody (RVNA) on Day 42 from start of the vaccination schedule

Other outcomes:

- Safety outcome measures will comprise of solicited post-vaccination reactions, vaccine-related SAEs, and unsolicited vaccine-related AEs at pre-defined time points from vaccination till 4 weeks after last vaccine dose.
- Geometric mean titres of Rabies Virus Neutralizing Antibody Concentrations on Day Days 14, 28, 90, 180 and 365 from start of the vaccination schedule
- Seroconversion rates (Rabies Virus Neutralizing Antibody Concentrations Equal and Above 0.5 IU/ml) on Days 14, 28, 42, 90, 180 and 365

12. **Safety evaluations**

Surveillance for AEs for the participants in the entire cohort will be carried out for each dose of vaccine and continued till 28 days after last dose as in vaccination schedule. Participants will be monitored for:

a) Immediate post-vaccination reactogenicity (within 30 min post-vaccination) after each dose of vaccine
b) Post-vaccination reactogenicity, i.e. the occurrence of solicited reactions after each dose of vaccination
c) occurrence of unsolicited adverse events after each dose in the vaccination schedule
d) Vaccine-related serious adverse events (SAEs), at any time throughout the entire duration of the study.

All SAEs that occur throughout the entire duration of the study will be reported to the Ethics Committee, following the required expedited procedures, independent of whether they are vaccine-related or not. All participating individuals will be closely followed to evaluate post-vaccination reactogenicity. For this purpose, the participants will be given a Post-Immunization Diary Card (PIDC). They will be instructed to record their highest axillary temperature as well as any solicited reactions and other AEs on PIDC during the interval between doses. The information on the solicited reactions and other AEs will also be collected when the participants come to take their scheduled dose or follow up visits. After the last dose of the vaccination schedule on Day 28, the participants will also be telephonically followed once in the month till the end of the study to inquire about the possible occurrence of other AEs and/or SAEs. Adverse events will be graded as mild, moderate and severe based on the DAIDS classification. The causality assessment of SAEs will be done using the standardised WHO-UMC system.

An external Data and Safety Monitoring Board (DSMB) will be set up for review of the safety data. We will conduct an initial meeting before the start of the study. The DSMB, will consist of independent vaccine and infectious diseases experts, including an internal medicine practitioner and a biostatistician, will periodically review cumulative data. The DSMB reviews will be summarized with recommendations to the study sponsors, as to whether or not there are safety concerns and whether the study should continue without change, be modified, or terminated. The sponsor will carefully consider DSMB recommendations. If a disagreement arises between the sponsor and the DSMB, the sponsor will discuss it with the DSMB in order to reach consensus. However, if attempts to reach consensus fail, the DSMB’s opinion will prevail. In such situation, the appropriate regulatory authorities will be informed.

An SAE is any untoward medical occurrence that:

- Results in death
- Is life threatening
- Requires inpatient hospitalization* or prolongation of existing hospitalization
- Results in persistent or significant disability**/incapacity
- Causes congenital anomaly/birth defect

13. **Sample size**

We plan to enrol 100 healthy volunteers in each of the four arms of the study. We assume equivalence hypothesis between two regimens, with power 90%, type I error 5%, true geometric mean ratio of 0.9, equivalence margin of 20%, (0.72 - 1.04) and coefficient of variation (COV) of 28%. We expect a dropout rate of 20%.

| **Assumptions** |  |
| --- | --- |
| True geometric mean ratio | 0.9 |
| Equivalence margin | 20% (0.72 - 1.04) |
| Coefficient of variation (COV) | 28% |
| Power | 90% |
| Type I error | 5% |
| Dropout rate | 20% |

14. **Randomization, sequence allocation and allocation concealment**

Healthy volunteers will be randomized into one of the four study arms. Permuted blocks of varying sizes of 4, 8 and 12 will be used to ensure balance between treatment regimens. A randomization list will be prepared using online available software (http://www.randomization.com) by ICMR-NIE. The allocation sequences will be generated before the study initiation, and allocation concealment will be managed by designing a secure android-based application to administer the allocation of interventions.

15. **Masking**

We will blind the personnel involved in laboratory testing on the nature of vaccine used.

16. **Data and sample collection**

The study participant will be assessed for eligibility to participate in the study. Consent in writing will be obtained. Information will be collected on basic demographic details, detailed medical history, history of exposure to animal bites and rabies immunization history. Physical examination will be done. information as per protocol would be collected for each volunteer using specific forms by a trained investigator using a tablet computer in an android based application. Once the study participant is included based on the assessments, a study serial number will be assigned. Participants will be randomized following a randomization list. The study regimen will be administered according to the assigned randomization number.

3-5 ml blood will be drawn on Days 0, 14, 28, 42, 90,180 and 365 for estimating rabies virus neutralizing antibody titres. Blood sample will be collected before each vaccine dose of the particular regimen. The samples will be coded at the trial site using the participant ID. The sera will be separated after centrifuging and will be stored at the ICMR-NIE. The sample will be periodically sent to NIMHANS for testing.

17. **Schedule of visits**

**The schedule of visits will be as follows**

The window period for the scheduled vaccination and blood sample collection is

given below. Between the second and third doses there should be a minimum gap of

three days.

| **Days of scheduled recruitment** | **Window period** |
| --- | --- |
| Day 0 | Not applicable |
| Day 3 | +1 day |
| Day 7 | +1 day |
| Day 14 | +1 day |
| Day 28 | +/- 2 days |
| Day 42 | +/- 2 days |
| Day 90 | +/- 2 days |
| Day 180 | +/- 2 days |
| Day 365 | +/- 2 days |

18. **Laboratory investigation**

A standard Rapid Fluoresecent Focus Inhibiton Test (RFFIT) will be used to estimate rabies virus neutralizing antibody titre. All the samples will be sent to ICMR-NIV for testing.

19. **Analysis Plan**

- The variables will be summarized using frequencies and percentages for categorical data, and mean with standard deviation or median with interquartile range for continuous data.
- Geometric mean titres of Rabies Virus Neutralizing Antibody Concentrations on Day 42 will be calculated for both regimens and Geometric Mean Ratio (GMR) will be estimated along with 95% CI. If the 95% Confidence limit of the observed GMR is within the range between 0.72 and 1.04, we will conclude that the immunogenicity is equivalent between two regimens.
- Geometric mean titres of Rabies Virus Neutralizing Antibody Concentrations, along with 95% CIon Days 14, 28, 42, 90,180 and 365 will be calculated and compared between the study groups.
- Proportion of Participants with seroconversion (Rabies Virus Neutralizing Antibody Concentrations Equal and Above 0.5 IU/ml) on Days 14, 28, 42, 90, 180 and 365 will be compared between the study groups using chi-square test.
- Proportion of participants with solicited post-vaccination reactions, vaccine-related SAEs, and unsolicited vaccine-related AEs will be profiled. Ninety-five percent confidence intervals will be provided by group using the Exact test. The SAEs will further be described by severity and in terms of individual listing.

20. **Ethics review:**

Approval from Ethics Committees of the ICMR-NIE and other participating institutions will be obtained. Study protocol, data collection tools, Participant Information sheet with informed consent forms in English and local language will be submitted to the Ethics Committee for approval. If a participant has been exposed to animal bite in between the study, he would be given the standard care and anti-rabies vaccine treatment as recommended by the National Rabies Control Programme of India (NRCP). We will ensure training and certification on ICH-GCP and Indian GCP of all the PIs involved before start of the study. We will register the trial with CTRI before commencement of the study. We will obtain clinical trial insurance for the participants. In case of any adverse event, the compensation to the participants will be given through the insurance company. The study sponsor will upfront pay a premium to cover insurance for the participants.

21. **Expected benefits:**

- The study will provide estimates of safety and immunogenicity of the Intradermal abridged Cambodia regimen with updated intradermal Thai red cross regimen in healthy volunteers in India
- The study will document evidence for safety and immunogenicity of the Intradermal abridged Cambodia regimen in Indian setting
- This might provide the evidence for the NRCP program mangers to implement WHO 2018 recommendations.

22. **Limitations of this study:**

The study is proposed to be conducted among healthy volunteers. Rabies virus can be immunoevasive, potentially altering the immunogenicity of the vaccine in presence of the virus which cannot be documented by the study. However, rabies virus neutralizing antibody concentration of 0·5 IU/mL or more at 14 days after immunisation is considered a proxy for protection in vaccine efficacy studies. Since the study involves healthy volunteers for anti-rabies vaccination and subsequent blood sample collection, drop out rates might be high.

23. **Schedule of Study Procedures**

During the study period, all the participants will visit the trial site as per the protocol-defined schedule of trial procedures.

| Study Visit | Day 0 | Day 3 | Day 7 | Day 14 | Day 28 | Day 42 | Day 90 | Day 180 | Day 365 |
| --- | --- | --- | --- | --- | --- | --- | --- | --- | --- |
| Informed consent | ✔ |  |  |  |  |  |  |  |  |
| Screening | ✔ |  |  |  |  |  |  |  |  |
| Clinical examination | ✔ | ✔ | ✔ |  | ✔ |  |  |  |  |
| Randomization | ✔ |  |  |  |  |  |  |  |  |
| Blood sample collection | ✔ |  |  | ✔ | ✔ | ✔ | ✔ | ✔ | ✔ |
| Vaccination | ✔ | ✔ | ✔ |  | ✔  (TRC regimen) |  |  |  |  |
| Post vaccination observation | ✔ | ✔ | ✔ |  | ✔ |  |  |  |  |
| Issue of diary card | ✔ |  |  |  |  |  |  |  |  |
| Review of Diary Card |  | ✔ | ✔ | ✔ | ✔ | ✔ |  |  |  |
| Digital thermometer and scale | ✔ |  |  |  |  |  |  |  |  |
| Follow up AE/SAEs |  | ✔ | ✔ | ✔ | ✔ | ✔ | ✔ | ✔ | ✔ |
| SAE monitoring |  | ✔ | ✔ | ✔ | ✔ | ✔ | ✔ | ✔ | ✔ |

**Reference:**

1. World Health Organization. WHO expert consultation on rabies: third report (accessed on 21^st^ February 2019)
2. Cleaveland S, Fevre EM, Kaare M, Coleman PG. Estimating human rabies mortality in the United Republic of Tanzania from dog bite injuries. Bulletin of the World Health Organization. 2002; 80(4):304-10.
3. Sudarshan MK, Narayana DH. Appraisal of surveillance of human rabies and animal bites in seven states of India. Indian J Public Health 2019;63, Suppl S1:3-8
4. Ramesh Masthi N R, Pradeep B S, Bilagumba G. A multicentric community survey on animal exposures among humans in India. Indian J Public Health 2019;63, Suppl S1:9-14
5. Cantaert T, Borand L, Kergoat L, et al. A 1-week intradermal dose-sparing regimen for rabies post-exposure prophylaxis (RESIST-2): an observational cohort study. Lancet Infect Dis. 2019;19(12):1355-1362. doi:10.1016/S1473-3099(19)30311-1
6. <https://www.cdc.gov/rabies/medical_care/index.html>
7. <https://ncdc.gov.in/WriteReadData/linkimages/GuidelinesforRabiesProphylaxis.pdf>
