# Supplementary material for: Immunogenicity and safety of Institut Pasteur du Cambodge (IPC) and updated Thai Red Cross (UTRC) intradermal rabies vaccine regimens among healthy adults in India: A phase 4, open label, equivalence randomised controlled trial
Source: PLoS Med. 2026 Oct 29;23(10):e1005079. doi: 10.1371/journal.pmed.1005079 (PMC13734584; doi:10.1371/journal.pmed.1005079)
Supplement: Supplementary file 8 [file pmed.1005079.s008.docx]

**S1 Checklist: CONSORT 2025 checklist of information to include when reporting a randomised trial***

| **Section / Topic** | **No** | **CONSORT 2025 checklist item description** | **Reported on page no.** |
| --- | --- | --- | --- |
| **Title and abstract** | | |  |
| Title and structured abstract | 1a | Identification as a randomised trial | Pp. 2 Title second sentence |
|  | 1b | Structured summary of the trial design, methods, results, and conclusions | Pp. 2,3 |
| **Open science** | | |  |
| Trial registration | 2 | Name of trial registry, identifying number (with URL) and date of registration | Pp. 3, Last Paragraph |
| Protocol and statistical analysis plan | 3 | Where the trial protocol and statistical analysis plan can be accessed | S7 Appendix |
| Data sharing | 4 | Where and how the individual de-identified participant data (including data dictionary), statistical code and any other materials can be accessed | Pp. 17 |
| Funding and conflicts of interest | 5a | Sources of funding and other support (e.g., supply of drugs), and role of funders in the design, conduct, analysis and reporting of the trial | Pp. 17 |
|  | 5b | Financial and other conflicts of interest of the manuscript authors | Pp. 17 |
| **Introduction** | | |  |
| Background and rationale | 6 | Scientific background and rationale | Para 4 (Introduction section) |
| Objectives | 7 | Specific objectives related to benefits and harms | Para 4 (Introduction section) |
| **Methods** | | |  |
| Patient and public involvement | 8 | Details of patient or public involvement in the design, conduct and reporting of the trial | Para 1 (Methods section) |
| Trial design | 9 | Description of trial design including type of trial (e.g., parallel group, crossover), allocation ratio, and framework (e.g., superiority, equivalence, non-inferiority, exploratory) | Para 1 (study design and participants section)  Para 1 (Randomization and masking section) |
| Changes to trial protocol | 10 | Important changes to the trial after it commenced including any outcomes or analyses that were not prespecified, with reason | Para 1 (study design and participants section)  Para 4 (Statistical analysis section) |
| Trial setting | 11 | Settings (e.g., community, hospital) and locations (e.g., countries, sites) where the trial was conducted | Para 1 and 2 (study design and participants section) |
| Eligibility criteria | 12a | Eligibility criteria for participants | Para 2 (study design and participants section) |
|  | 12b | If applicable, eligibility criteria for sites and for individuals delivering the interventions (e.g., surgeons, physiotherapists | Para 2 (study design and participants section) |
| Intervention and comparator | 13 | Intervention and comparator with sufficient details to allow replication. If relevant, where additional materials describing the intervention and comparator (e.g., intervention manual) can be accessed | Para 1. (procedures section)  Para 1 (Estimation of RVNA titres) |
| Outcomes | 14 | Pre-specified primary and secondary outcomes, including the specific measurement variable (e.g., systolic blood pressure), analysis metric (e.g., change from baseline, final value, time to event), method of aggregation (e.g., median, proportion), and time point for each outcome | Para 1(Outcomes section |
| Harms | 15 | How harms were defined and assessed (e.g., systematically, non-systematically) | Para 3 and 4(Procedures section) |
| Sample size | 16a | How sample size was determined, including all assumptions supporting the sample size calculation | Para 1 (Statistical analysis |
|  | 16b | Explanation of any interim analyses and stopping guidelines | Nil |
| Randomisation: |  |  |  |
| Sequence generation | 17a | Who generated the random allocation sequence and the method used | Para 2 (Randomization and masking section) |
|  | 17b | Type of randomisation and details of any restriction (e.g., stratification, blocking and block size) | Para 1 (Randomization and masking section) |
| Allocation concealment mechanism | 18 | Mechanism used to implement the random allocation sequence (e.g., central computer/telephone; sequentially numbered, opaque, sealed containers), describing any steps to conceal the sequence until interventions were assigned | Para 2 (Randomization and masking section) |
| Implementation | 19 | Whether the personnel who enrolled and those who assigned participants to the interventions had access to the random allocation sequence | Para 2 (Randomization and masking section) |
| Blinding | 20a | Who was blinded after assignment to interventions (e.g., participants, care providers, outcome assessors, data analysts) | Para 2 (Randomization and masking section) |
|  | 20b | If blinded, how blinding was achieved and description of the similarity of interventions | Para 2 (Randomization and masking section) |
| Statistical methods | 21a | Statistical methods used to compare groups for primary and secondary outcomes, including harms | Para 2 & 3 (Statistical analysis section) |
|  | 21b | Definition of who is included in each analysis (e.g., all randomised participants), and in which group | Para 2 & 3 (Statistical analysis section) |
|  | 21c | How missing data were handled in the analysis | Para 2 & 3 (Statistical analysis section) |
|  | 21d | Methods for any additional analyses (e.g., subgroup and sensitivity analyses), distinguishing prespecified from post-hoc | Para 4 (Statistical analysis) |
| **Results** | | |  |
| Participant flow, including flow diagram | 22a | For each group, the numbers of participants who were randomly assigned, received intended intervention, and were analysed for the primary outcome | Figure 1 study flow diagram; S3 Appendix |
|  | 22b | For each group, losses and exclusions after randomisation, together with reasons | Figure 1 study flow diagram; S3 Appendix |
| Recruitment | 23a | Dates defining the periods of recruitment and follow-up for outcomes of benefits and harms | Para 1 (Procedures section) |
|  | 23b | If relevant, why the trial ended or was stopped | Nil |
| Intervention and comparator delivery | 24a | Intervention and comparator as they were actually administered (e.g., where appropriate, who delivered the intervention/comparator, how participants adhered, whether they were delivered as intended [fidelity]) | Para 1 (Results section) Figure 1 study flow diagram; |
|  | 24b | Concomitant care received during the trial for each group | Nil |
| Baseline data | 25 | A table showing baseline demographic and clinical characteristics for each group | Table 1 |
| Numbers analysed,  outcomes and estimation | 26 | For each primary and secondary outcome, by group:   - the number of participants included in the analysis - the number of participants with available data at the outcome time point - result for each group, and the estimated effect size and its precision (such as 95% confidence interval) - for binary outcomes, presentation of both absolute and relative effect size | Para 3,4 &5 (Results section) Tables 2 and 3 |
| Harms | 27 | All harms or unintended events in each group | Para 6 (Results section) |
| Ancillary analyses | 28 | Any other analyses performed, including subgroup and sensitivity analyses, distinguishing pre-specified from post-hoc | Para 5 (Results section) Tables 2 and 3 |
| **Discussion** | | |  |
| Interpretation | 29 | Interpretation consistent with results, balancing benefits and harms, and considering other relevant evidence | Para 1 and 9 (Discussion section) |
| Limitations | 30 | Trial limitations, addressing sources of potential bias, imprecision, generalisability, and, if relevant, multiplicity of analyses | Para 8 (Discussion section) |

*We strongly recommend reading this statement in conjunction with the CONSORT 2025 Explanation and Elaboration and/or the CONSORT 2025 Expanded Checklist for important clarifications on all the items. We also recommend reading relevant CONSORT extensions. See [www.consort-spirit.org](http://www.consort-spirit.org).

Citation: Hopewell S, Chan AW, Collins GS, Hróbjartsson A, Moher D, Schulz KF, et al. CONSORT 2025 Statement: updated guideline for reporting randomised trials. BMJ. 2025; 388:e081123. <https://dx.doi.org/10.1136/bmj-2024-081123>.

© 2025 Hopewell et al. This is an Open Access article distributed under the terms of the Creative Commons Attribution License (<https://creativecommons.org/licenses/by/4.0/>), which permits unrestricted use, distribution, and reproduction in any medium, provided the original work is properly cited

**Reporting of Noninferiority and Equivalence Randomized Trials: Extension of the CONSORT 2010 Statement***

| **Section/Topic** | **Item No.** | **Standard CONSORT 2010 Checklist Item** | **Extension for Noninferiority/Equivalence Trials** | **Page no; Paragraph and section** |
| --- | --- | --- | --- | --- |
| **Title and abstract** | 1a | Identification as a randomised trial in the title. | *Identification as a noninferiority or equivalence randomised trial in the title.* | Page no 2 Title second sentence |
|  | 1b | Structured summary of trial design, methods, results, and conclusions (for specific guidance see CONSORT for abstracts). | *See separate Abstract Checklist (Table 2) for noninferiority/equivalence trials.* | Page no. 2,3 |
| **Introduction** | 2a | Scientific background and explanation of rationale. | *Rationale for using a noninferiority or equivalence design.* | Para 4 (Introduction section) |
|  | 2b | Specific objectives or hypotheses. | *Hypotheses concerning noninferiority or equivalence, specifying the noninferiority/equivalence margin with the rationale for its choice.* | Para 4 (Introduction section) |
| **Methods** | 3a | Description of trial design (such as parallel, factorial) including allocation ratio. | No modification to the standard CONSORT 2010 item. | Para 1 (study design and participants section)  Para 1 (Randomization and masking section) |
|  | 3b | Important changes to methods after trial commencement (such as eligibility criteria), with reasons. | No modification to the standard CONSORT 2010 item. | Para 1 (study design and participants section) |
|  | 4a | Eligibility criteria for participants. | *Whether participants in the noninferiority/equivalence trial are similar to those in any trial(s) that established efficacy of the reference treatment.* | Para 2 (study design and participants section) |
|  | 4b | Settings and locations where the data were collected. | No modification to the standard CONSORT 2010 item. | Para 1 &2 (Procedures section) |
|  | 5 | The interventions for each group with sufficient details to allow replication, including how and when they were actually administered. | *Whether the reference treatment in the noninferiority/equivalence trial is identical (or very similar) to that in any trial(s) that established efficacy.* | Para 1 (Procedures section) |
|  | 6a | Completely defined pre-specified primary and secondary outcome measures, including how and when they were assessed. | *Specify the noninferiority/equivalence outcome(s) and whether hypotheses for main and secondary outcome(s) are noninferiority, equivalence, or superiority. Whether the outcomes are identical (or very similar) to those in trial(s) that established efficacy of the reference treatment.* | Para 1(Outcomes section) |
|  | 6b | Any changes to trial outcomes after the trial commenced, with reasons. | No modification to the standard CONSORT 2010 item. | Nil |
|  | 7a | How sample size was determined. | *Whether the sample size was calculated using a noninferiority or equivalence criterion and, if so, what the margin was.* | Para 1 (Statistical analysis section) |
|  | 7b | When applicable, explanation of any interim analyses and stopping guidelines. | *To which outcome(s) interim analyses/stopping rules apply, and whether related to a noninferiority or equivalence hypothesis.* | Nil |
|  | 8a | Method used to generate the random allocation sequence. | No modification to the standard CONSORT 2010 item. | Para 1 (Randomization and masking section) |
|  | 8b | Type of randomisation; details of any restriction (such as blocking and block size). | No modification to the standard CONSORT 2010 item. | Para 1 (Randomization and masking section) |
|  | 9 | Mechanism used to implement the random allocation sequence, describing steps taken to conceal the sequence until interventions were assigned. | No modification to the standard CONSORT 2010 item. | Para 2 (Randomization and masking section) |
|  | 10 | Who generated the random allocation sequence, who enrolled participants, and who assigned participants to interventions. | No modification to the standard CONSORT 2010 item. | Para 2 (Randomization and masking section) |
|  | 11a | If done, who was blinded after assignment to interventions and how. | No modification to the standard CONSORT 2010 item. | Para 2 (Randomization and masking section) |
|  | 11b | If relevant, description of the similarity of interventions. | No modification to the standard CONSORT 2010 item. | Nil |
|  | 12a | Statistical methods used to compare groups for primary and secondary outcomes. | *Whether a 1-sided or 2-sided confidence interval approach was used.* | Para 2 & 3 (Statistical analysis section) |
|  | 12b | Methods for additional analyses, such as subgroup analyses and adjusted analyses. | No modification to the standard CONSORT 2010 item. | Para 4 (Statistical analysis section) |
| **Results** | 13a | For each group, the numbers of participants who were randomly assigned, received intended treatment, and were analysed for the primary outcome. | No modification to the standard CONSORT 2010 item. | Para 1 (Under the results section) Figure 1 study flow diagram; S3 Appendix |
|  | 13b | For each group, losses and exclusions after randomisation, together with reasons. | No modification to the standard CONSORT 2010 item. | Figure 1 study flow diagram; S3 Appendix |
|  | 14a | Dates defining the periods of recruitment and follow-up. | No modification to the standard CONSORT 2010 item. | Para 1 (Procedures section) |
|  | 14b | Why the trial ended or was stopped. | No modification to the standard CONSORT 2010 item. | Nil |
|  | 15 | A table showing baseline demographic and clinical characteristics for each group. | No modification to the standard CONSORT 2010 item. | Para 2 (Results section) Table 1 |
|  | 16 | For each group, number of participants included in each analysis and whether the analysis was by original assigned groups. | No modification to the standard CONSORT 2010 item. | Figure 1 study flow diagram; S3 Appendix |
|  | 17a | For each primary and secondary outcome, results for each group, and the estimated effect size and its precision (such as 95% confidence interval). | *For the outcome(s) for which noninferiority or equivalence was hypothesised, a figure showing confidence intervals and the noninferiority/equivalence margin may be useful.* | Para 3,4 &5 (Results section) |
|  | 17b | For binary outcomes, presentation of both absolute and relative effect sizes is recommended. | No modification to the standard CONSORT 2010 item. | Para 3 (Results section) Tables 2 and 3 |
|  | 18 | Results of any other analyses performed, including subgroup analyses and adjusted analyses, distinguishing pre-specified from exploratory. | No modification to the standard CONSORT 2010 item. | Para 5 (Results section) Tables 2 and 3 |
|  | 19 | All important harms or unintended effects in each group (for specific guidance see CONSORT for harms). | No modification to the standard CONSORT 2010 item. | Para 6 (Results section) Tables 5 and 6 |
| **Discussion** | 20 | Trial limitations, addressing sources of potential bias, imprecision, and, if relevant, multiplicity of analyses. | No modification to the standard CONSORT 2010 item. | Para 8 (Discussion section) |
|  | 21 | Generalisability (external validity, applicability) of the trial findings. | No modification to the standard CONSORT 2010 item. | Para 2,3, &4 (Discussion section) |
|  | 22 | Interpretation consistent with results, balancing benefits and harms, and considering other relevant evidence. | *Interpret results in relation to the noninferiority or equivalence hypothesis. If a superiority conclusion is drawn for outcome(s) for which noninferiority/equivalence was hypothesised, provide justification for switching.* | Para 1and 9 (Discussion section) |
| **Other information** | 23 | Registration number and name of trial registry. | No modification to the standard CONSORT 2010 item. | Para 1 (Study design and participants) |
|  | 24 | Where the full trial protocol can be accessed, if available. | No modification to the standard CONSORT 2010 item. | Provided as s8 appendix |
|  | 25 | Sources of funding and other support (such as supply of drugs), role of funders. | No modification to the standard CONSORT 2010 item. | Para 1 (competing interests)  Para 1 (Funding) |

*Citation: Piaggio G, Elbourne DR, Pocock SJ, Evans SJW, Altman DG, for the CONSORT Group. Reporting of Noninferiority and Equivalence Randomized Trials: Extension of the CONSORT 2010 Statement. JAMA. 2012;308(24):2594–2604. doi:10.1001/jama.2012.87802.

Abstract of a Report of a Noninferiority or Equivalence Randomized Trial: Extension of CONSORT for Abstracts Checklist*

| **Item** | **Standard Checklist Item** | **Extension for Noninferiority/Equivalence Trials** | **Page no** |
| --- | --- | --- | --- |
| **Title** | Identification of study as randomised. | *Identification of study as a noninferiority or equivalence trial.* | Page no 2 Title |
| **Trial design** | Description of the trial design (e.g., parallel, cluster, noninferiority). | — | Page no 2, second paragraph  (Under methods and findings section) |
| **Participants** | Eligibility criteria for participants and the settings in which the data were collected. | — | Page no 2, second and third paragraphs  (Under methods and findings section) |
| **Interventions** | Interventions intended for each group. | — | Page no 2, second paragraph  (Under methods and findings section) |
| **Objective** | Specific objective or hypothesis. | *Specific hypothesis concerning noninferiority or equivalence, including the margin.* | Page no 2, first paragraph  (Under background section)  Page no 2, second paragraph  (Under methods and findings section) |
| **Outcome** | Clearly defined primary outcome for this report. | *Clarify for all reported outcomes whether noninferiority, equivalence, or superiority is being assessed.* | Page no 2, second paragraph  (Under methods and findings section) |
| **Randomisation** | How participants were allocated to interventions. | — | Page no 2, second and third paragraphs  (Under methods and findings section) |
| **Blinding (masking)** | Whether participants, caregivers, and those assessing the outcomes were blinded to group assignment. | — | Page no 2, second paragraph  (Under methods and findings section |
| **Numbers randomised** | Number of participants randomised to each group. | — | Page no 2, third paragraph  (Under methods and findings section) |
| **Recruitment** | Trial status. | — | Page no 2, third paragraph  (Under methods and findings section) |
| **Numbers analysed** | Number of participants analysed in each group. | — | Page no 2, third paragraph  (Under methods and findings section) |
| **Outcome** | For the primary outcome, a result for each group and the estimated effect size and its precision. | *For the primary noninferiority/equivalence outcome, results in relation to the margin.* | Page no 2, third paragraph  (Under methods and findings section) |
| **Harms** | Important adverse events or side effects. | — | Page no 2, third paragraph  (Under methods and findings section) |
| **Conclusions** | General interpretation of the results. | *Interpretation taking into account the noninferiority/equivalence hypotheses and any superiority hypotheses.* | Page no 3, first paragraph  (Under conclusion section) |
| **Trial registration** | Registration number and name of trial register. | — | Page no 3, third paragraph  (Under trial registration section) |
| **Funding** | Source of funding. | — | Nil |

*Citation: Piaggio G, Elbourne DR, Pocock SJ, Evans SJW, Altman DG, for the CONSORT Group. Reporting of Noninferiority and Equivalence Randomized Trials: Extension of the CONSORT 2010 Statement. JAMA. 2012;308(24):2594–2604. doi:10.1001/jama.2012.87802
